# Comparison of the difference in the anti-inflammatory activity of two different color types of Farfarae Flos based on *in vitro*, *in vivo* experiments and untargeted metabolomics

**DOI:** 10.3389/fphar.2024.1463864

**Published:** 2024-09-24

**Authors:** Kexin Zhou, Liang Peng, Yiyao Jing, Yao Luo, Yonggang Yan, Gang Zhang, Qi Guo, Bingyue Yang

**Affiliations:** ^1^ Shaanxi Qinling Application Development and Engineering Center of Chinese Herbal Medicine, College of Pharmacy, Shaanxi University of Chinese Medicine, Xi’an, China; ^2^ Key Laboratory for Research of “Qin Medicine” of Shaanxi Administration of Traditional Chinese Medicine, Xi’an, China

**Keywords:** Farfarae Flos, anti-inflammatory, non-targeted metabolomics, interleukin-6 (IL-6) inhibition, nitric oxide (NO) inhibition

## Abstract

**Introduction:**

Due to its remarkable anti-inflammatory pharmacological activity, Farfarae Flos has gained extensive usage in the treatment of various inflammatory diseases such as bronchitis, pneumonia, prostatitis and colitis. And Farfarae Flos come in two color types depending on the color of the flowers: yellowish-white (YW), and purplish-red (PR). However, the difference in anti-inflammatory activity and metabolic profiles between the two flower colors remains unexplored.

**Methods:**

This study aims to explore the difference in the anti-inflammatory potential between YW and PR variants of Farfarae Flos and unravel the mechanisms responsible for the observed differences in anti-inflammatory activity through an integrated approach encompassing untargeted metabolomics and *in vivo*/*vitro* experimental studies. Initially, we verified the contrasting effects of YW and PR on the inhibition of the inflammatory factors interleukin-6 (IL-6) and nitric oxide (NO) by utilizing an *in vitro* RAW 264.7 cell inflammation model. Subsequently, a comprehensive evaluation of the systemic inhibitory capacity of YW and PR on IL-6, Interleukin-10 (IL-10), and tumor necrosis factor-*α* (TNF-*α*) was conducted using a validated whole-body mouse model, followed by the analysis of inflammatory factors and histological examination of collected serum, liver, and spleen after 7 days. Furthermore, non-targeted metabolomics profiling was employed to analyze the metabolite profiles of Farfarae Flos with different colors, and quantitative analysis was conducted to identify differential metabolites between YW and PR. The correlation between the anti-inflammatory activities of differentially accumulated metabolites (DAMs) and Farfarae Flos was investigated, resulting in the identification of 48 compounds exhibiting significant anti-inflammatory activity. Additionally, KEGG pathway enrichment analysis was performed to elucidate the underlying mechanisms.

**Results:**

Our findings demonstrate that both YW and PR possess anti-inflammatory abilities, with PR exhibiting significantly superior efficacy. The integration of *in vivo*/*vitro* experiments and non-targeted metabolomics confirmed the exceptional anti-inflammatory potential of PR and solidified its classification as the “purplish-red better” of Farfarae Flos.

**Discussion:**

This study provides valuable insights into the breeding and medical transformation of Farfarae Flos varieties, along with a scientific basis for the establishment of quality standards and the development of new drugs utilizing Farfarae Flos.

## Graphical Abstract

**Figure:**
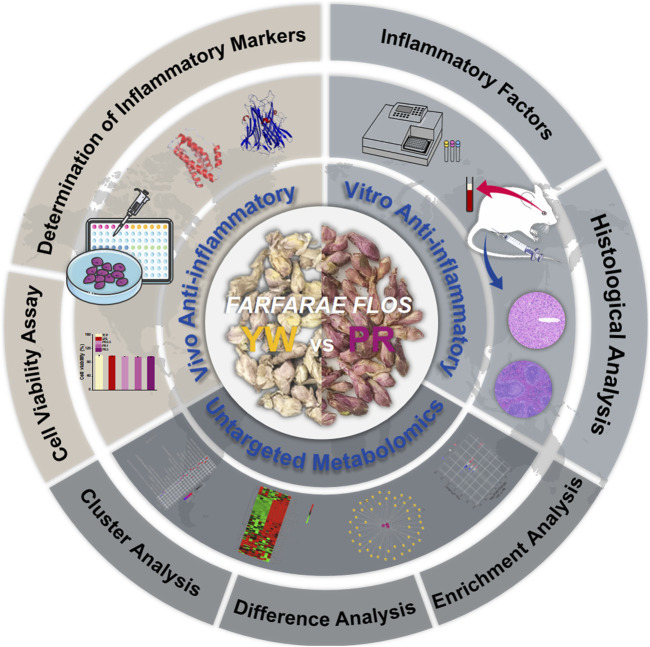

## 1 Introduction

Inflammation, a fundamental pathological process triggered by internal or external stimuli, is categorized into acute and chronic inflammation (Kany et al., 2019). Acute inflammation, usually resolves spontaneously, while chronic inflammation is characterized by excessive production of inflammatory factors such as nitric oxide (NO), tumor necrosis factor-*α* (TNF-*α*), and interleukin-6 (IL-6) (Kapellos et al., 2019; Tanaka et al., 2020). Inflammatory responses are closely associated with various diseases, including cardiovascular, metabolic diseases, neurodegenerative disorders, and other inflammatory conditions such as chronic gastritis, rheumatoid arthritis, inflammatory bowel disease, and cancer (Pan et al., 2021; Sugimoto et al., 2019). Although a moderate inflammatory response is beneficial for the body’s defense against harmful stimuli, excessive or prolonged inflammation can lead to numerous detrimental effects, including fever, redness, tumors, pain and loss of function. In fact, in clinical settings, widely employed anti-inflammatory medications like aspirin and ibuprofen are frequently linked to side effects and allergic responses (Bellón, 2019; Braun et al., 2020; Moore et al., 2019).

In contrast to Western medicines, the use of herbal medicine is steeped in ancient history, with its origins tracing back over millennia (Nunes et al., 2020; Shin et al., 2020). Today, natural and herbal substances continue to receive increasing attention due to their low side effects and cost-effectiveness (Yimer et al., 2019). In recent years, the anti-inflammatory effects of herbal medicine have been extensively studied. Numerous *in vivo*/*vitro* studies have demonstrated that herbal medicine exerts its anti-inflammatory effects by impeding key transcription factors, mitigating the activity of pro-inflammatory cytokines and chemokines, reducing intercellular adhesion molecule expression, and inhibiting the action of pro-inflammatory mediators (Li et al., 2020; Tasneem et al., 2019; Xin et al., 2019).

**FIGURE 1:**
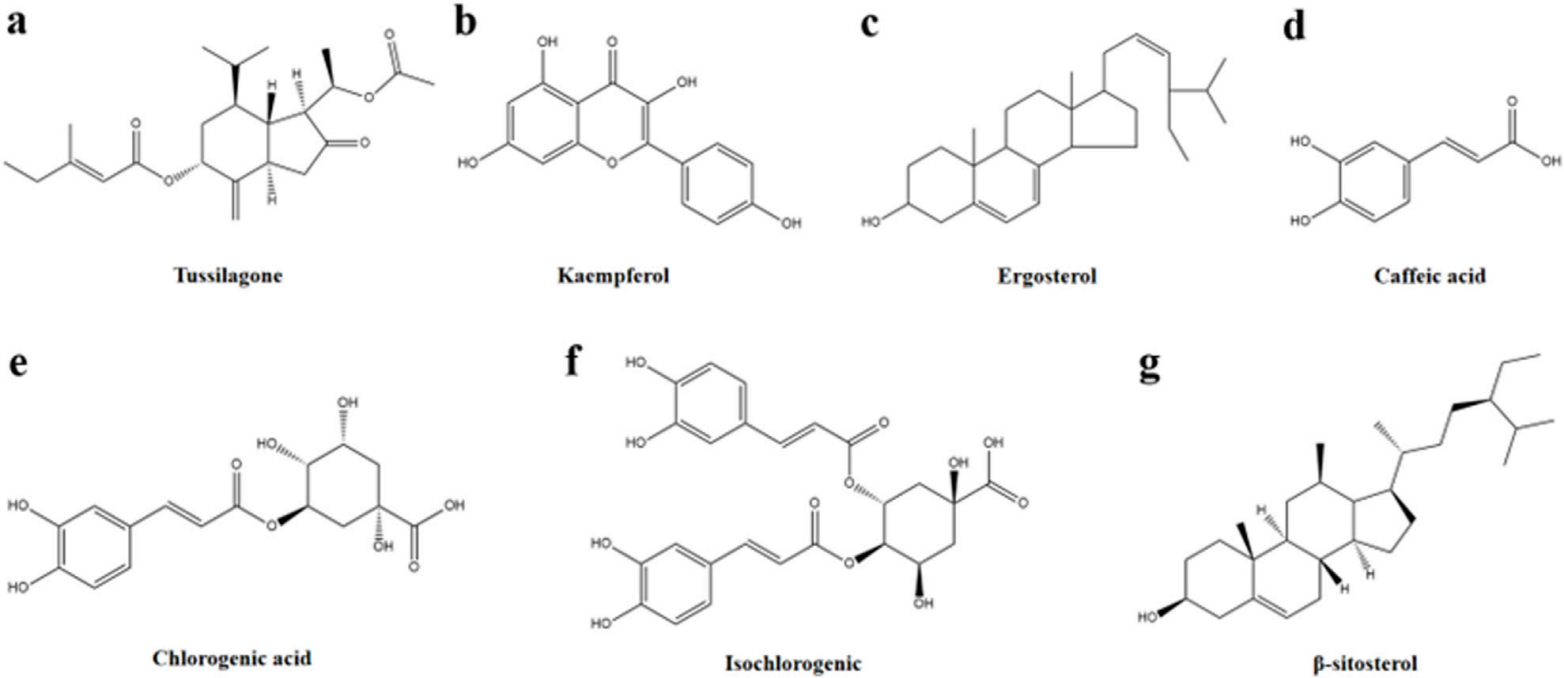
Chemical structures of compounds **(A–G)**.

*Tussilago farfara* L., the only plant in the genus *Tussilago* L. in the family Asteraceae, is widely distributed across Asia, Europe and North Africa. The flower buds of *T. farfara* L. commonly known as Farfarae Flos, have long been used in traditional medicine to moisten the lungs, alleviate coughs, reduce phlegm, and lower airway inflammation (Xuan et al., 2020). Moreover, Farfarae Flos exhibits notable anti-inflammatory, anti-allergic, and anti-platelet aggregation properties (Liu et al., 2020). In China, the Farfarae Flos has been used in the form of dried flower buds from ancient times to the present day, and it is used in Chinese medicine as one of the essential medicines for relieving coughs and phlegm and asthma (Jang et al., 2019). In some European countries, indigenous people also use Farfarae Flos to treat coughs and colds. Nevertheless, they are more likely to use the leaves of the herb for the treatment of diseases of the gastrointestinal tract, wounds, burns, urinary and inflammatory damage in the eyes (Kalle et al., 2022). In recent years, Farfarae Flos and related compound preparations have displayed significant therapeutic effects on a wide range of inflammatory conditions, making it a critical species in the development of anti-inflammatory drugs. Numerous studies have identified various compounds in Farfarae Flos, including flavonoids, sterols, phenolic acids, alkaloids, polysaccharides, volatile oils, sesquiterpenoids, triterpenoids, and other bioactive components. The anti-inflammatory effects of key active monomeric components such as sesquiterpenoids (e.g., Tussilagone), flavonoids (e.g., kaempferol), sterols (e.g., ergosterol and β-sitosterol), and organic acids (e.g., chlorogenic acid, isochlorogenic acid, and caffeic acid) have been clinically validated (Chen et al., 2021; Ferrer et al., 2018; Jin et al., 2020; Song et al., 2019; Yang et al., 2020) (Figure 1).

It is well known that Farfarae Flos is dominated by two different colors, purplish-red (PR) and yellowish-white (YW), and the difference between the two colors is obvious and can be easily judged by visual performance. The difference in color often corresponds to variations in pharmacological activity (Zou et al., 2021). Traditionally, the criterion of “purplish-red better” has been employed to evaluate the quality of Farfarae Flos. However, precise studies explaining the regularity behind the superior anti-inflammatory activity of PR compared to YW are lacking. Hence, it is crucial to compare the anti-inflammatory activity between these two colors and conduct an in-depth investigation into the main components and material basis underlying the difference in their anti-inflammatory activity.

In this study, the difference in anti-inflammatory effects between YW and PR through *in vitro* experiments was initially determined. Furthermore, the anti-inflammatory effects of YW and PR were evaluated using a mouse systemic inflammation model, accompanied by histological analysis. Additionally, non-targeted metabolomics analysis was performed to assess the natural differences in metabolite types between YW and PR, and to investigate the reasons for the difference. The presence of differentially accumulated metabolites (DAMs) of YW and PR were annotated into the KEGG pathway, revealing that phenylpropanoid, flavonoid and flavonol, and sesquiterpene metabolism pathways were strongly associated with the anti-inflammatory activity of Farfarae Flos. Moreover, the correlation between anti-inflammatory activities and highly differentially accumulated metabolites (DAMs) of YW and PR was examined. A total of 48 compounds, including flavonoids, sesquiterpenes, sterols, and other compound types, exhibited high correlations with anti-inflammatory activities. These results underscore the correlation between the build up of secondary metabolites and the varying degrees of anti-inflammatory efficacy observed between YW and PR. In conclusion, this study provides comprehensive insights into the patterns and mechanisms underlying the contrasting anti-inflammatory effects of different colors of Farfarae Flos, offering valuable guidance for selecting Farfarae Flos varieties and facilitating its clinical application.

## 2 Experimental

### 2.1 Preparation of Farfarae Flos

**FIGURE 2:**
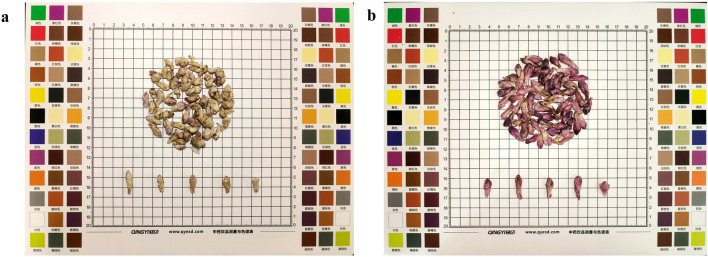
**(A)** A sample of dried YW of Farfarae Flos buds; **(B)** A sample of dried PR of Farfarae Flos buds.

Farfarae Flos was purchased from Shaanxi Best Enterprise Group. The herb material was identified by Prof. Hu Benxiang. The voucher specimens of Farfarae Flos were preserved in the herbarium of Shaanxi University of Chinese Medicine. All samples were divided into two groups, yellow-white (YW) (Figure 2A) and purplish-red (PR) (Figure 2B), using the color card as a control. Both groups were dried, crushed, and sieved through 40 mesh (pore size 0.425 mm), placed in paper bags and stored in a desiccator at room temperature.

### 2.2 Chemical solvents

All chemical solvents information can be found in Supplementary Material.

### 2.3 Preparation of extracts

Took 80 g of each kind of powder from purplish-red and yellowish-white Farfarae Flos, extracted with 85% ethanol for 1 h and filtered while hot, the residue was rinsed three times, concentrated under reduced pressure at 56°C to obtain the extracts of different groups. The extracts were dissolved in distilled water at established concentrations and were recorded as PR for the purplish-red flowers and YW for the yellowish-white flowers.

### 2.4 Biocompatibility test of YW and PR

Biocompatibility of YW and PR was evaluated by RAW 264.7 cells (Johnson et al., 1983). The cell viabilities of YW and PR were evaluated by alamarBlue^®^ assay. First, RAW 264.7 cells underwent standard thawing procedures and were grown in a humidified incubator containing 5% CO_2_ at 37°C for 24 h. The cells were passaged, and after the cells grown to 80% of their maximum density, RAW 264.7 cells were seeded in a 96-well plate at a density of 10,000 cells/well. After being cultured for 24 h, complete mediums containing YW and PR at different concentrations and colors were introduced into the wells. The details of the cytocompatibility evaluation were obtained in the Supplementary Material.

### 2.5 Animal research

All animals (Kunming mice; 20 ± 2 g; half male and female) were domesticated in the animal laboratory for 7 days before the start of the animal tests. All animal experiments were conducted by the “Regulations on the Administration of Experimental Animals,” the Chinese Animal Experiment Guidelines, and internationally accepted ethical principles for the use and care of experimental animals. The animal experiments involved in this study have been approved by the ethics committee (approval number: SUCMDL20220725001).

### 2.6 NO production assay

RAW 264.7 cells were seeded into 96-well plates at a density of 10,000 cells per well and incubated for 24 h. Subsequently, complete mediums containing YW and PR were introduced into the wells. After being co-incubated for another 24 h, cell suspensions were centrifuged and the cell culture supernatant was obtained. The NO content in the cell culture supernatant was determined by a commercial nitric oxide (NO) content assay. All operations strictly follow the manufacturer’s instructions. Optical density (OD) readings of the samples were taken at 530 nm using a microplate reader to determine the NO levels in the supernatant.

### 2.7 ELISA test

ELISA kits were utilized to quantify the secretion levels of NO, TNF-*α*, IL-10, and IL-6 in RAW 264.7 cells induced by LPS. Initially, RAW 264.7 cells were plated at a seeding in 96-well plates and allowed to adhere for 12 h. Following attachment, the cells were treated with LPS at a concentration of 1 μg/mL and incubated together for 24 h. After incubation, the cell suspension was harvested and subjected to centrifugation at 1,200 rpm to separate the cell culture supernatant. The concentrations of NO, TNF-*α*, IL-10, and IL-6 in the supernatant were then determined according to the protocols provided with the ELISA kits. The assay was designed to permit up to 10 replicate measurements for each sample.

### 2.8 Anti-inflammatory test

After 7 days of adaptation, 90 KM mice were randomly assigned into 9 different groups (n = 10) randomly, including the TCP (tissue culture plate, means this group does not undergo any treatment) group (H_2_O), LPS group (H_2_O), positive group (Diclofenac Sodium 20 mg/kg), HYW group (High-dose group of YW), MYW group (Middle-dose group of YW), LYW group (Low-dose group of YW), HPR group (High-dose group of PR), MPR group (Middle-dose group of PR) and LPR group (Low-dose group of PR). The dosage of the above experimental groups: high-dose group (5 g/kg), medium-dose group (2.5 g/kg), low-dose group (1.25 g/kg), and all the dosages administered were equivalent to the crude drug. The dose was administered daily at 9:00 a.m. for 7 days. 2 h after the last dose, LPS (5 mg/kg) was injected intraperitoneally into the LPS and dosing groups, and 200 µL of PBS was injected into the TCP (without treatment) group. 2 h later, all mice were anesthetized, blood was taken from the eyes, and the serum was collected after immediate centrifugation. After the blood sampling was completed, the liver and spleen were quickly removed and preserved in a 4% paraformaldehyde solution.

### 2.9 Histological analysis

Histological analysis was performed by H&E staining. The livers and spleens of the mice were cleaned with saline, preserved in 4% paraformaldehyde, embedded in paraffin, and then sectioned. These sections were subsequently stained using Hematoxylin and Eosin (H&E) and observed and photographed using a microscope.

### 2.10 Metabolite extraction and UPLC-MS/MS analysis for the untargeted metabolomic analysis

Five samples of about 1 g each were taken from each of the two groups of Farfarae Flos, labeled yellowish-white 1–5 for the YW and purplish-red group 1–5 for the PR, wrapped in tin foil and stored in liquid nitrogen for quick-freezing until use. Take the appropriate amount of samples (50 mg) in a 2 mL EP tube, according to the sample processing method of metabolomics, the 5 groups of samples of YW and PR were further processed, and the detailed processing information, metabolic sample preparation and UPLC MS/MS chromatographic data can be found in the Supplementary Material.

### 2.11 Bioinformatic analysis of the untargeted metabolomic dataset

The bioinformatic examination of the untargeted metabolomic dataset was conducted using the XCMS and ProteoWizard software suites, alongside the CAMERA and metaX toolboxes, all these tools were implemented by using R software (see Supplementary Material for further details). The annotation of metabolites was facilitated through the use of the online KEGG and HMDB databases. In addition, an in-house library of metabolite fragment profiles was used to validate metabolite identification.

## 3 Results

### 3.1 Biocompatibility of YW and PR

**FIGURE 3:**
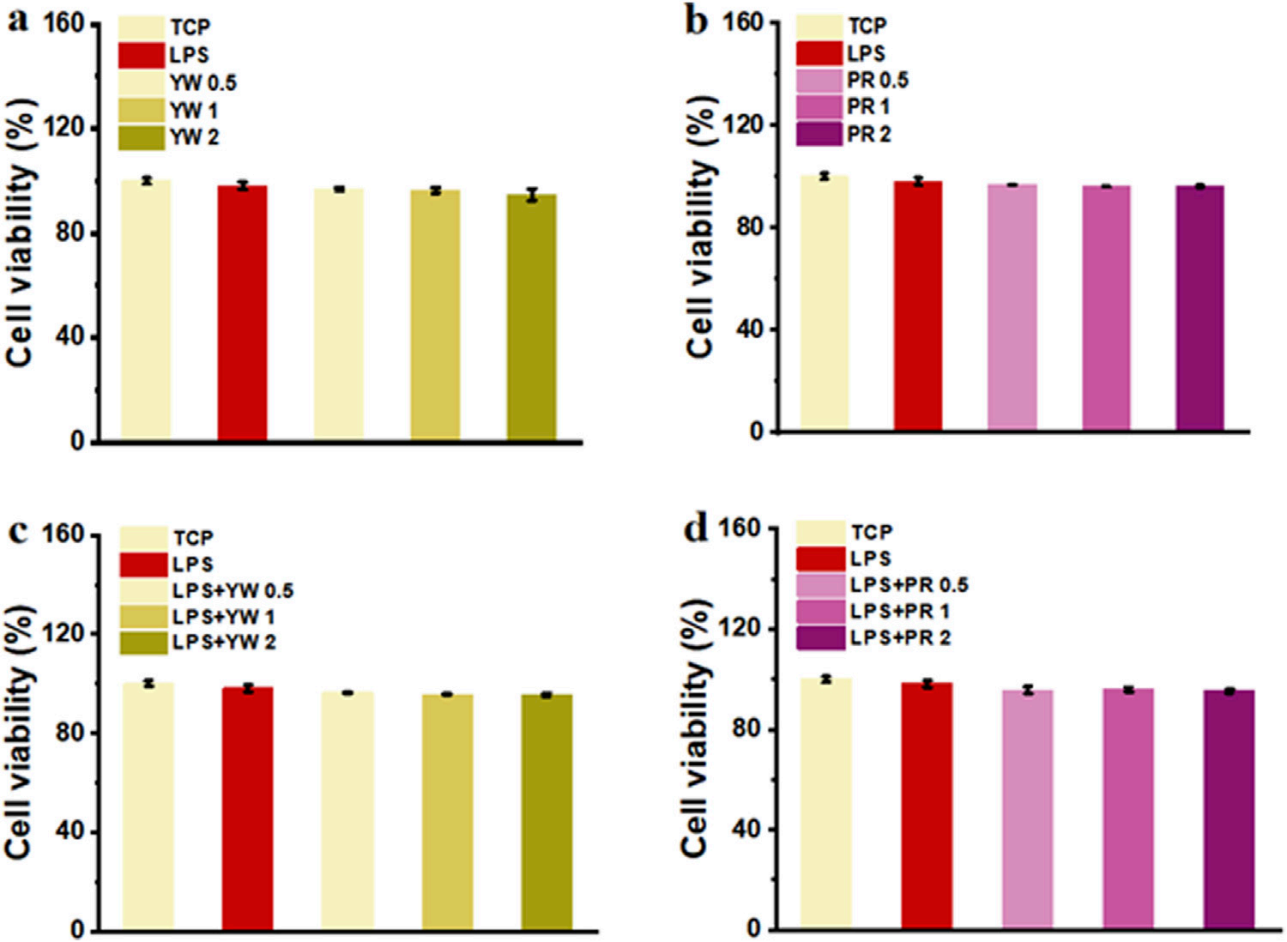
**(A)** Cell viability of YW from various groups; **(B)** Cell viability of PR from various groups; **(C)** Cell viability of YW with LPS treatment from various groups; **(D)** Cell viability of PR with LPS treatment from various groups.

Good cytocompatibility is the premise for YW and PR to achieve the medicinal effect. In this study, the biocompatibility of YW and PR was assessed by evaluating the cell viability of RAW 264.7 cells after treatment with these extracts *in vitro*. After 24 h of treatment with complete mediums containing YW and PR, the cell viability of all groups exceeded 90% (Figures 3A–D), indicating that the cell viability was not significantly inhibited by YW and PR treatment. Although the cell viability slightly decreased with increasing concentrations of YW and PR, there were no significant differences compared to the TCP group. Notably, the cell viability of RAW 264.7 cells remained above 90% even at an extract concentration of 2 mg/mL. Additionally, to exclude the impact of lipopolysaccharide (LPS)-induced polarization of RAW 264.7 cells, the cell viability of LPS-treated cells was tested after 24 h. Similarly, the reduction in cell viability after LPS treatment was negligible. Moreover, the cell viability after treatment with YW, PR, and LPS was comparable to that of the TCP group. In conclusion, YW and PR demonstrated favorable cytocompatibility.

### 3.2 Anti-inflammatory activity of YW and PR

**FIGURE 4:**
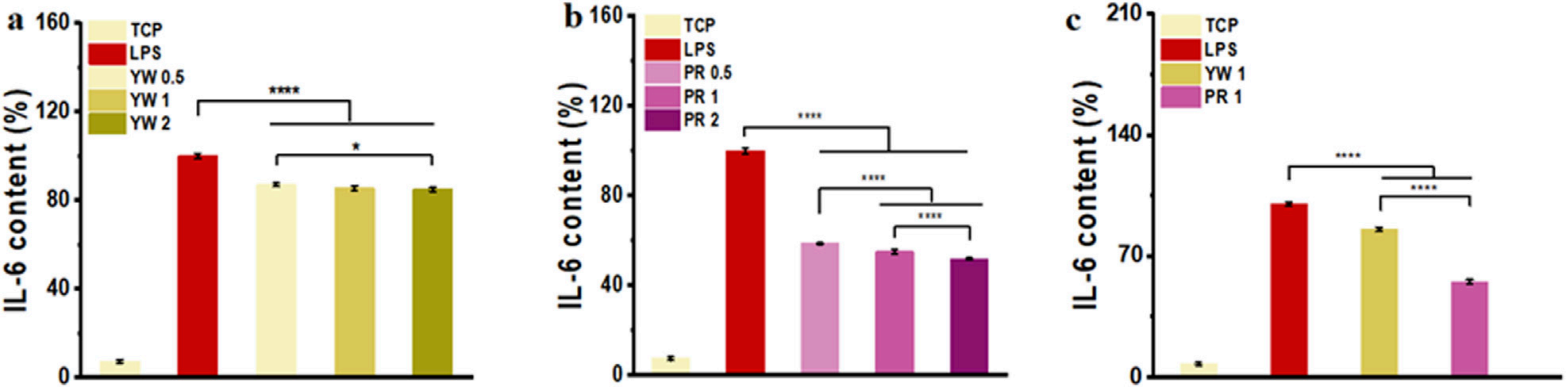
IL-6 content in the supernatant of RAW 264.7 cells treated with YW **(A)**, PR **(B)** at 0.5 mg/mL, 1 mg/mL and 2 mg/mL, IL-6 content in the supernatant of RAW 264.7 cells treated with YW and PR **(C)** at 1 mg/mL. (^*^ *p* < 0.05, ^**^ *p* < 0.01, ^***^ *p* < 0.001, ^****^ *p* < 0.0001).

As a cytokine mediating inflammatory immune response, IL-6 plays a critical role in the body’s anti-infection immune response. To further confirm the inhibitory effect of Farfarae Flos extracts on inflammation, we evaluated the intracellular IL-6 levels of YW and PR after LPS treatment. IL-6 levels were significantly increased in RAW 264.7 cells after LPS treatment. As shown in Figures 4A–C, both YW and PR significantly reduced intracellular IL-6 levels compared to the LPS-treated group (*p* < 0.0001). The high-dose (2 mg/mL) groups of YW and PR exhibited significantly lower IL-6 levels compared to the medium-dose (1 mg/mL) group and low-dose (0.5 mg/mL) groups of YW and PR, indicating a dose-dependent increase in the anti-inflammatory capacity of both YW and PR. With the increase of the administered concentration, the IL-6 content in the cells showed a negative increase and the cellular inflammatory response was diminished. Most importantly, at the same concentration of YW, PR demonstrated a significantly greater reduction in IL-6 levels, highlighting its superior anti-inflammatory activity compared to YW (*p* < 0.0001).

**FIGURE 5:**
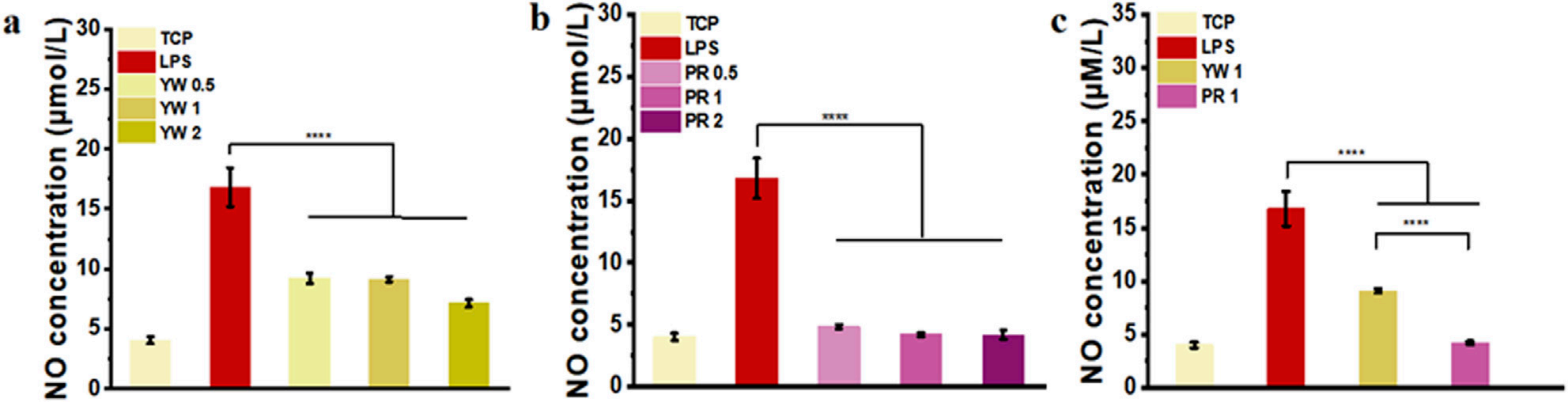
NO content in the supernatant of RAW 264.7 cells treated with **(A)** YW and **(B)** PR at concentrations of 0.5 mg/mL, 1 mg/mL and 2 mg/mL, respectively; **(C)** NO concentration in the supernatant of RAW 264.7 cells treated with YW and PR at concentrations of 1 mg/mL. (^****^ *p* < 0.0001).

Nitric oxide (NO) serves as a signaling molecule with inflammatory properties, regulating various physiological activities. In this study, the ability of YW and PR to scavenge NO was evaluated to verify their anti-inflammatory effects. As illustrated in Figures 5A–C, the LPS-treated group significantly induced NO production in RAW 264.7 cells compared to the TCP group. Compared to the LPS-treated group, both YW and PR were able to reduce the NO concentration significantly (*p* < 0.0001). At concentrations of 0.5 mg/mL, 1 mg/mL, and 2 mg/mL, YW reduced the NO concentration to 9.2 μmol/L, 9.1 μmol/L, and 7.1 μmol/L, respectively, which were significantly lower than the NO concentration in the LPS-treated group (16.8 μmol/L) (Figure 5A). PR also exhibited a dose-dependent increase in the inhibitory effect on NO concentration, with the high-dose group of PR reducing NO concentration to 4.18 μmol/L (Figure 5B), which was not statistically different comparable the TCP group (4.03 μmol/L). Furthermore, a significant difference was observed between YW and PR at high, medium, and low doses (*p* < 0.05). Additionally, when comparing the intracellular NO scavenging abilities of YW and PR at a concentration of 1 mg/mL, PR exhibited significantly stronger NO scavenging ability than YW (*p* < 0.0001, Figure 5C). These results confirm that PR had the best NO scavenging capacity, and it is worth mentioning that the reduced NO levels after administration via the high-dose group of PR could even reach the NO levels of the TCP group. Obviously, both YW and PR demonstrated desirable inhibitory effects on inflammation, with PR exhibiting superior anti-inflammatory activity compared to YW.

### 3.3 Therapeutic effects of the YW and PR on the model of systemic inflammation

**FIGURE 6:**
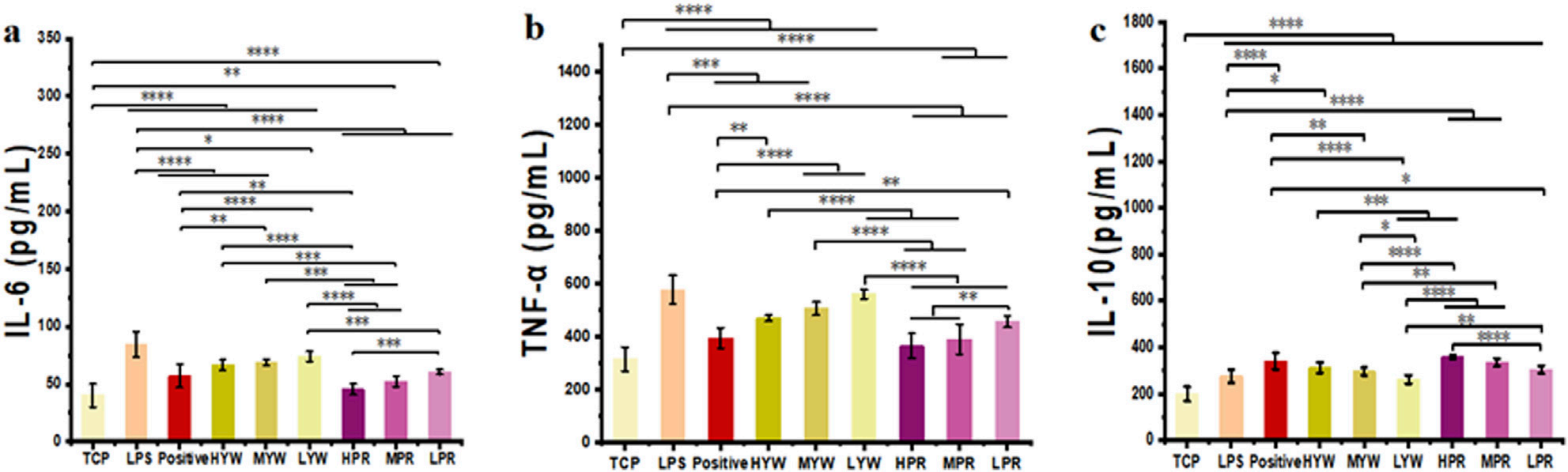
Content of **(A)** IL-6, **(B)** TNF-*α* and **(C)** IL-10 in mouse serum of TCP, control group, positive group and YW and PR (n = 10). (^*^ *p* < 0.05, ^**^ *p* < 0.01, ^***^ *p* < 0.001, ^****^ *p* < 0.0001).

Lipopolysaccharide (LPS) is a major component of bacterial outer membranes that causes inflammatory responses in disease and low doses of LPS in mice can induce systemic inflammation. After LPS treatment, the pro-inflammatory cytokines in the mice will increase in a short time. Therefore, to investigate the anti-inflammatory potential of Farfarae Flos, LPS was used to induce systemic acute inflammation in mice. Serum samples from different treatment groups were collected to measure the release of inflammatory cytokines IL-6, TNF-*α*, and IL-10, serving as indicators of the anti-inflammatory activity of YW and PR. We compared the inhibition of inflammatory factors in LPS-treated mice by YW and PR using mice administered without drugs as the TCP group, mice administered with LPS only as the control group and mice administered with Diclofenac Sodium Sustained Release Tablets as the positive group, as shown in Figures 6A–C. Quantitative data show that in comparison to the LPS control group, Administration of YW and PR at all tested doses suppressed the secretion of the pro-inflammatory cytokines IL-6 and TNF-*α* in mice. Likewise, all doses of YW and PR promoted the release of the anti-inflammatory cytokine IL-10. Notably, the release of IL-6, IL-10 and TNF-*α* in PR significantly differed from that in the TCP, control, positive and YW groups (*p* < 0.0001). In comparison to the control, positive control and high-dose group of YW, PR reduced the release of pro-inflammatory cytokine IL-6 by 46.29%, 20.33%, and 31.9%, respectively. The release of pro-inflammatory cytokine TNF-*α* decreased by 36.58%, 7.64%, and 22.16% correspondingly. Additionally, the expression of the anti-inflammatory factor IL-10 increased by 29.63%, 5.3%, and 14.86%. Consistent with the results in Section 3.2, the ability of YW and PR to regulate inflammatory factors in mouse serum improved with increasing doses. Moreover, PR demonstrated a superior anti-inflammatory effect on LPS-induced systemic inflammation in mice compared to YW, with the high-dose group of PR even outperforming the positive group. These results align with the findings of previous *in vitro* experiments. Hence, PR exhibits notable anti-inflammatory activity and holds significant therapeutic and clinical application potential. Further investigation is necessary at the molecular level to elucidate any differences in the anti-inflammatory activity between YW and PR and the underlying mechanisms.

### 3.4 Histological analysis

**FIGURE 7:**
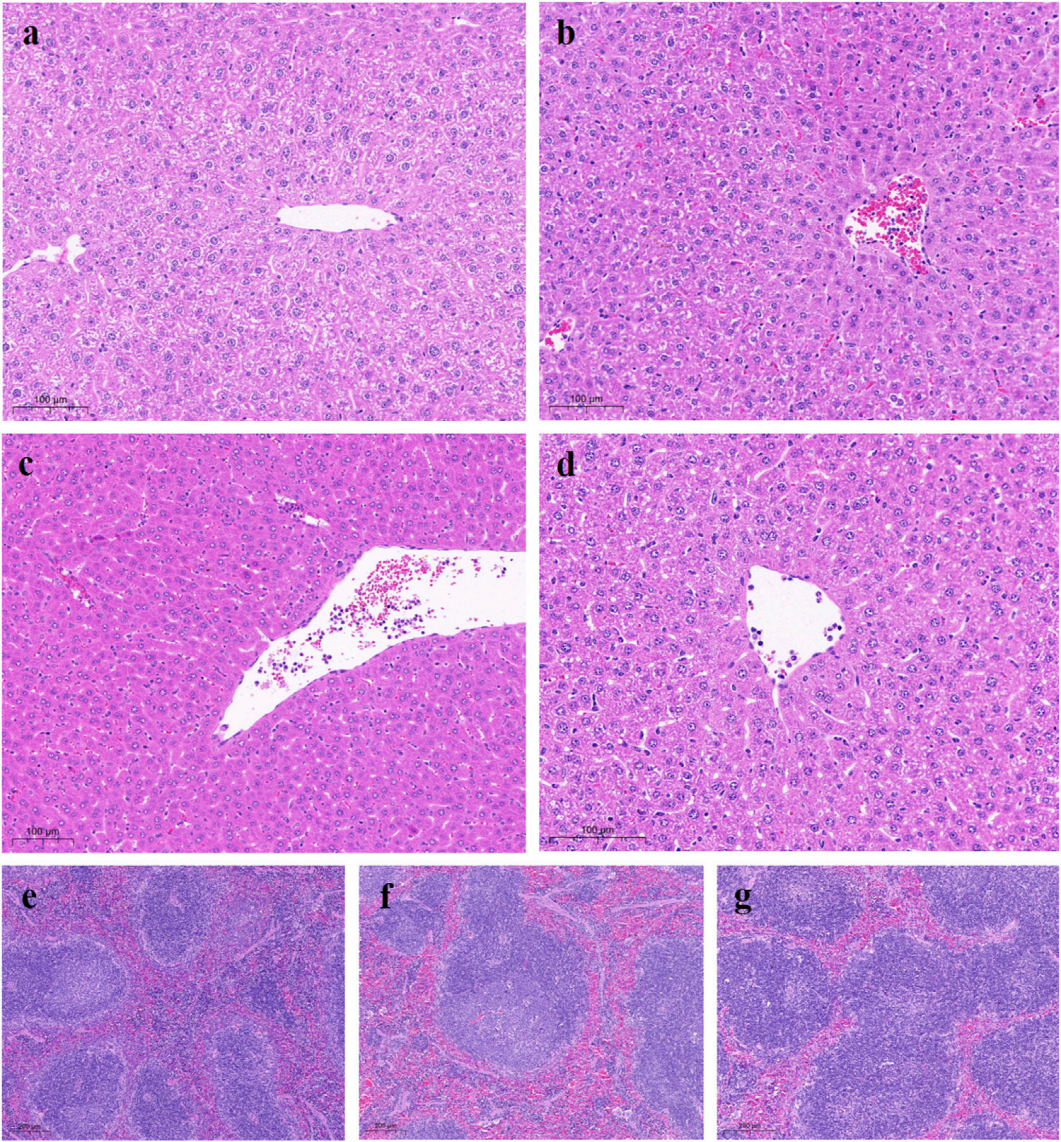
Representative microscopic images of H&E stained liver sections from all study groups. **(A)** Liver TCP group: liver showing normal tissue of hepatocytes and hepatic lobules; **(B)** Control group: mice liver with hepatocytes, most of which had cytoplasmic vacuoles; **(C)** High-dose group of YW: gradual recovery of hepatocytes; **(D)** High-dose group of PR: hepatic lobule structure was clearly restored; **(E)** Spleen TCP group; **(F)** Spleen positive group; **(G)** High-dose group of PR.

Histological sections of normal liver tissues showed normal lobular structure with central veins and radial hepatic cords accompanied by intact hepatocytes with a homogeneous distribution of cytoplasm. The control group showed marked morphological changes and fibrosis, as indicated by disruption of the histological structure, fibrous extension, formation of large fibrous septa, pseudobulbar separation and fibrous accumulation. Additionally, substantial hepatocyte degeneration, necrosis, hepatic sinusoidal dilatation, inflammatory cell infiltration, and cytoplasmic vacuole formation were observed. Treatment with YW and PR resulted in noticeable improvements in liver structure, as evidenced by reduced hepatic injury, inhibited hepatocyte degeneration and necrosis, and significant restoration of liver structure and function (Figure 7). Importantly, PR exhibited better protection against liver and spleen injury compared to YW.

### 3.5 Difference in metabolic components between YW and PR analyzed by UPLC-MS/MS

Our findings revealed that both YW and PR exhibited anti-inflammatory effects. However, PR demonstrated significantly superior activity compared to YW. Notably, the high-dose group of PR displayed the highest level of anti-inflammatory activity. Despite these results, the underlying mechanism responsible for the difference in activity between YW and PR remains elusive. To gain further insight into the anti-inflammatory activity of Farfarae Flos at the molecular level, we employed an untargeted metabolomics approach to analyze the metabolite profiles of YW and PR.

#### 3.5.1 Principal component analysis (PCA)

**FIGURE 8:**
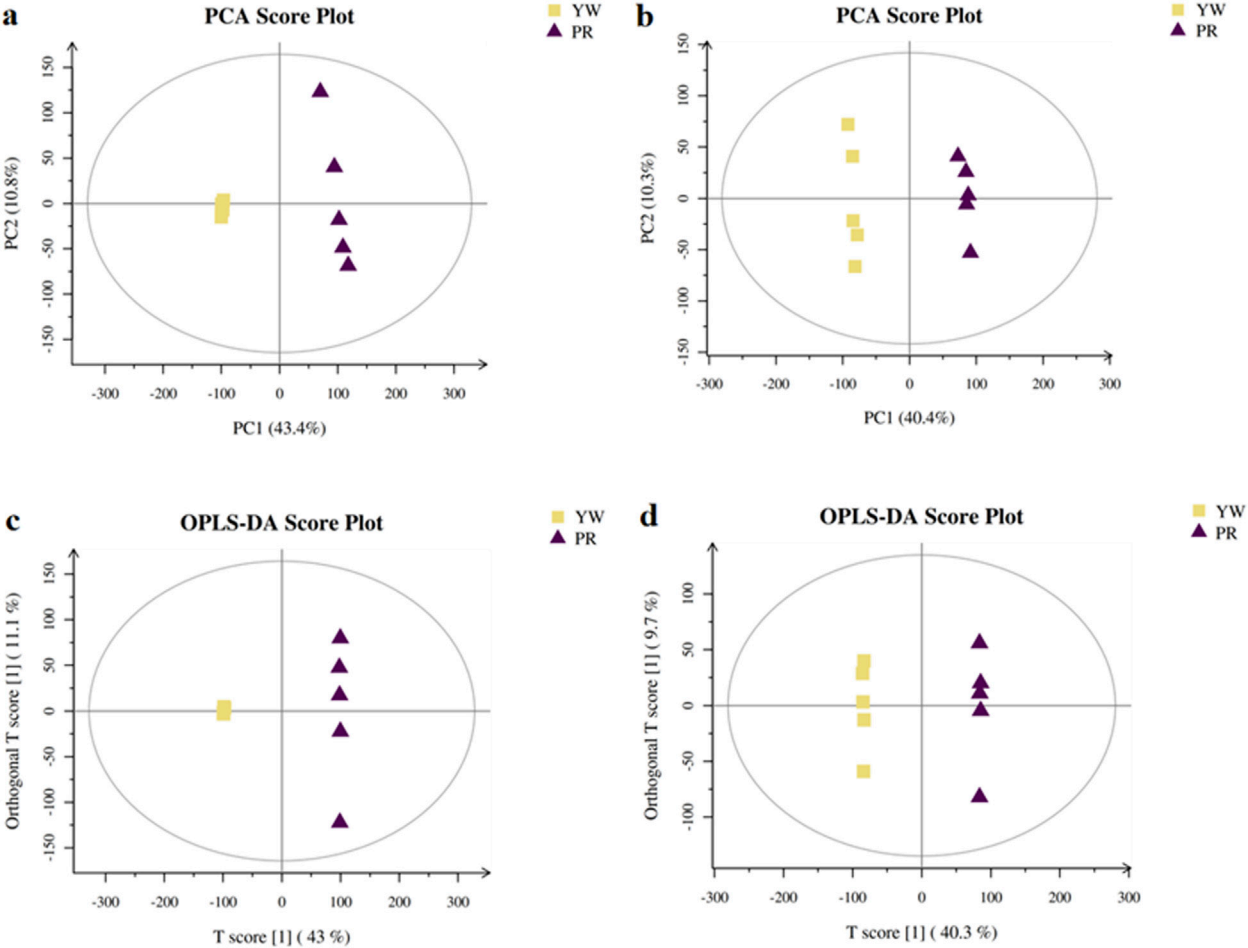
PCA scores plot of YW and PR in positive ion **(A)** and negative ion **(B)**; The OPLS-DA score plots in the positive ion mode **(C)** and negative ion mode **(D)**.

In this study, we investigated the metabolic differences between YW and PR using UPLC-MS/MS analysis. First, we performed unsupervised pattern recognition principal component analysis (PCA) to compare the overall distribution trends of secondary metabolites and assess whether there were any differences between YW and PR. As depicted in Figures 8A, B, the PCA 2D plots clearly showed two distinct clusters corresponding to YW and PR based on their spectral features. PR mainly clustered along the positive axis of PC1, while YW predominantly clustered along the negative axis of PC1. The coefficient of variation (CV) values were below 30%, indicating good reproducibility. In both positive and negative ion modes, PC1 and PC2 accounted for a significant amount of the variation, demonstrating that YW and PR could be easily differentiated. There was no overlap between the YW and PR clusters, suggesting substantial differences in metabolite profiles between the two groups. Thus, the PCA analysis confirmed a significant distinction in the metabolites of YW and PR.

#### 3.5.2 Orthogonal partial least squares discriminant analysis (OPLS-DA)

To delve deeper into the metabolic distinctions between YW and PR, we used OPLS-DA as our analytical method. High predictability (Q^2^) is a key parameter representing the predictive ability of the model, when Q^2^ > 0.9 and the goodness of fit is strong, the OPLS-DA analytical model as well as the VIP values (Variable Importance Projections) generated by the model are stable, reliable and valid. As shown in Figures 8C, D, the Q^2^ value of 0.957 indicated a clear separation between YW and PR metabolites, confirming a distinct trend of separation in their metabolite profiles.

### 3.6 Screening of differential metabolites, functional annotation, and enrichment analysis between the groups

**FIGURE 9:**
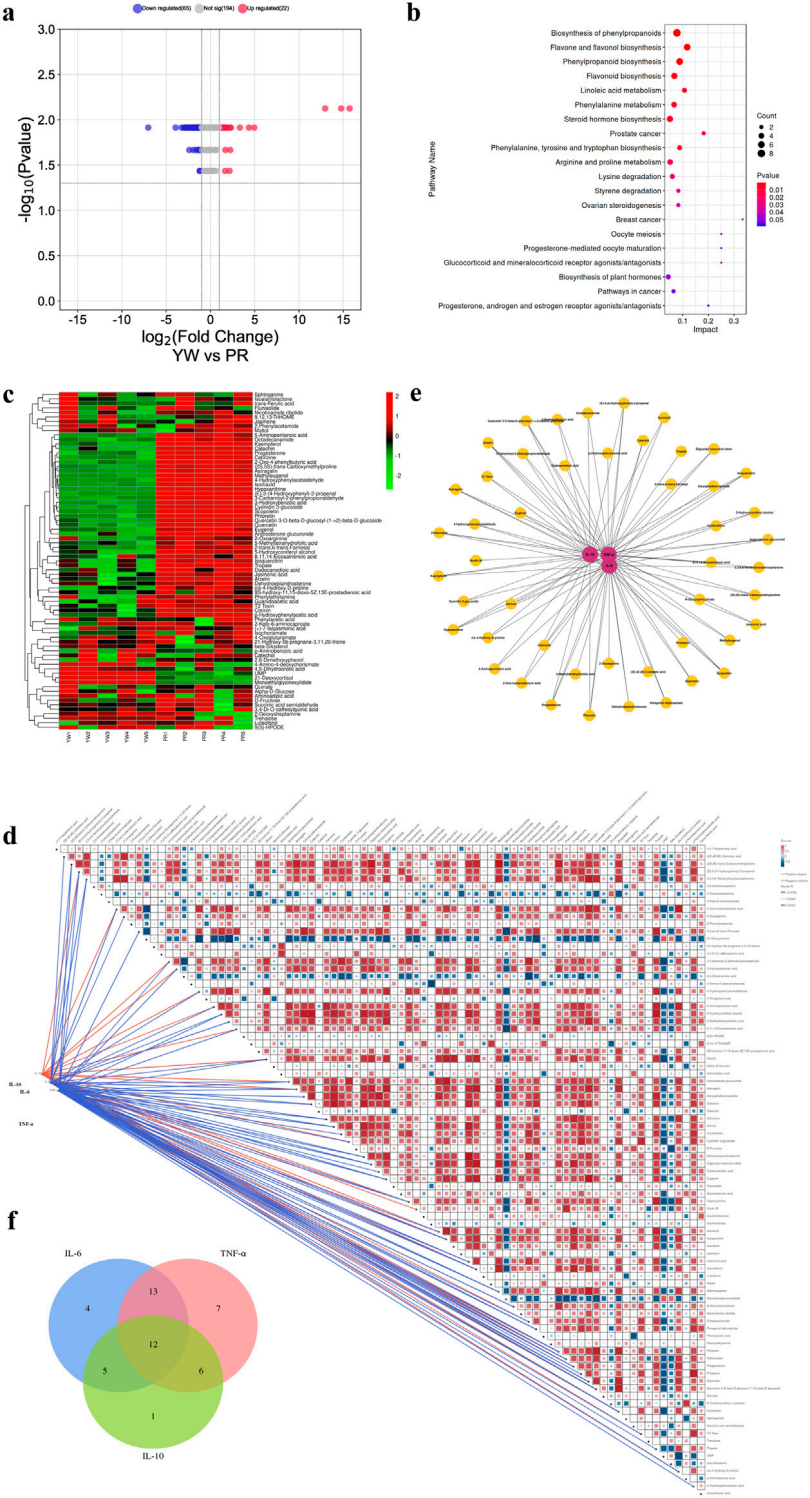
**(A)** Volcano plot of differential metabolites between YW and PR. Blue indicates downregulation, red indicates upregulation; **(B)** Pathway map of the top 20 KEGG metabolites annotated by DAMs; **(C)** Clustering heatmap of 87 DAMS. Green indicates downregulation, red indicates upregulation; **(D)** Correlation network diagram of 87 DAMs with three inflammatory factors (IL-6, TNF-*α*, IL-10); **(E)** Network map between metabolites and anti-inflammatory capacity. Red circles indicate different inflammatory factors, yellow circles indicate different metabolites, and the line connecting two circles indicates correlation (r ≥ 0.7, p < 0.028); **(F)** Correlation of metabolites with three inflammatory factors in the Venn plot.

Next, we conducted differential metabolite screening, functional annotation, and enrichment analysis to identify the most representative differential metabolites between YW and PR. Using UPLC-MS/MS qualitative and quantitative analysis, we identified 281 differential metabolites in YW and PR (VIP values ≥1, Fold change ≥2, *p* ≤ 0.05). Compared with PR, the 87 DAMs in the samples of YW changed significantly. The volcano plot provided an overview of the distribution of differentially expressed metabolites, showcasing upregulated metabolites highlighted in red and downregulated metabolites depicted in blue, which can also serve as the functional analysis of metabolic pathways. As shown in Figure 9A, among the differential metabolites, 22 metabolites upregulated and 65 metabolites downregulated. These changed metabolites included flavonoids, alkaloids, phenolic acids, nucleotides, and their derivatives.

To gain insight into the biological functions associated with these differential metabolites, we performed KEGG metabolic pathway enrichment analysis using Metabo Analyst 5.0. The top 20 metabolic pathways enriched are represented as enrichment bubble plots as shown in Figure 9B.

### 3.7 Correlation analysis of the secondary metabolites in Farfarae Flos and their anti-inflammatory activity

Furthermore, we examined the correlation between the 87 DAMs and the anti-inflammatory activities (IL-6, TNF-*α*, and IL-10) to gain further insight into the anti-inflammatory components present in Farfarae Flos by using Spearman correlation coefficients. The clustering heatmap (Figure 9C) and correlation network diagram (Figure 9D) depicted the relationships between the 87 differential metabolites and the three inflammatory factors (IL-6, TNF-*α*, and IL-10). We identified 48 metabolites that were significantly correlated with anti-inflammatory activities (|r| ≥ 0.7, p < 0.028), covering various compound types such as alkaloids, flavonoids, sterols, sesquiterpenes, coumarins, and others (Figure 9E). Analysis using Spearman correlation indicated that there was a positive correlation between 24 distinct metabolites and the anti-inflammatory cytokine IL-10. Conversely, 47 distinct metabolites showed a negative correlation with the pro-inflammatory cytokines IL-6 and TNF-*α*. Additionally, 12 differential metabolites showed correlations with all three inflammatory factors (Figure 9F). Table 1 provides the names and classifications of these 12 metabolites, including six flavonoids and flavonols, one coumarin, one sterol, one sesquiterpene, and three other compounds. Among them, Quercetin exhibited the highest correlation coefficient with IL-6 (|r| = 0.977), N-Glucosylnicotinate showed the highest correlation coefficient with TNF-*α* (|r| = 0.966), and the flavonoids Quercetin 3-O-beta-D-glucosyl-(1->2)-beta-D-glucoside, Afzelin, and Isoquercitrin displayed the highest correlation coefficients with IL-10 (r = 0.830). Based on the correlation analysis and multiple factors, we speculate that the differential content of these compounds may contribute to the observed differences in the anti-inflammatory capacity and pharmacological effects between YW and PR. In addition to flavonoids, alkaloids, and sesquiterpenes, coumarins (e.g., Scopoletin) and sterols (e.g., Progesterone) may also play significant roles as anti-inflammatory substances in Farfarae Flos.

**TABLE 1:** 12 DAMs were screened out that are closely related to inflammatory factors.

Number	Compounds	Classifications
1	Quercetin 3-O-beta-D-glucosyl-(1->2)-beta-D-glucoside	Flavonoids and Flavonols
2	Phloretin	
3	Kaempferol	
4	Isoquercitrin	
5	Astragalin	
6	Afzelin	
7	Scopoletin	Coumarins
8	Progesterone	Sterols
9	Polhovolide	Sesquiterpenes
10	Hypoxanthine	Others
11	(E)-3-(4-Hydroxyphenyl)-2-propenal	
12	Androsterone glucuronide	

## 4 Discussion

In recent years, the identification and quality control of medicinal flowers have remained crucial in botanical medicine research (Hadizadeh et al., 2022; Jeyaraj et al., 2022). Farfarae Flos has emerged as a valuable resource for anti-inflammatory drug development due to its rich content of various anti-inflammatory active ingredients such as flavonoids, sesquiterpenoids, and sterols (Yang et al., 2022). However, there is still a need for further exploration of its anti-inflammatory potential. The lack of uniform evaluation criteria for medication selection in clinics and limited understanding of the differences in anti-inflammatory activity between the two most common colors, yellowish-white (YW) and purplish-red (PR), necessitate further investigation. Although previous studies have explored that Farfarae Flos exerts anti-inflammatory effects through the regulation of Nrf2, NF-κB, and NLRP3 inflammasome (Aralbaeva et al., 2017; Kim et al., 2006; Xu et al., 2022), the underlying mechanisms contributing to the differential anti-inflammatory activity of different colors based on secondary metabolomics remain unexplored. Hence, to standardize the clinical use of Farfarae Flos and ensure efficacy and safety, there is an urgent need to investigate the variation in anti-inflammatory activity between YW and PR and understand the potential underlying mechanisms from the perspective of secondary metabolite accumulation.

In this study, we established a cellular inflammation model by inducing RAW 264.7 macrophages with lipopolysaccharide (LPS). The results demonstrated that both YW and PR effectively inhibit the release of inflammatory factors IL-6 and NO, and indicating initial differences in anti-inflammatory effects between the two variants. Furthermore, in an experimental model of systemic inflammation in mice, we observed that both YW and PR suppressed the release of pro-inflammatory cytokines IL-6 and TNF-*α* while promoting the release of the anti-inflammatory factor IL-10 in the serum of mice. Histological analysis confirmed that both YW and PR ameliorated liver and spleen damage induced by systemic inflammation. Notably, all of these results proved that PR consistently exhibited superior anti-inflammatory effects compared to YW, but the mechanisms underlying these differences in activity have not been found.

The metabolomics approach provided a valuable opportunity for a comprehensive investigation into the metabolic variations exhibited by Farfarae Flos collected at different stages, raw and processed Farfarae Flos (Cao et al., 2021; Li et al., 2018; Li et al., 2017). Therefore, we employed an untargeted metabolomic approach to analyze the metabolite profiles of YW and PR, aiming to further elucidate the anti-inflammatory activities of Farfarae Flos at the molecular level. The PCA and OPLS-DA results showed a large population difference and high separation between YW and PR, which further verified that there were significant anti-inflammatory activity differences between YW and PR in terms of characterization. Meanwhile, we also screened all the differential metabolites in YW and PR and found 281 differential metabolites. Screening these metabolites with VIP values ≥1, Fold change ≥2, *p* ≤ 0.05, 22 metabolites displayed elevated levels in YW compared to PR, while 65 metabolites exhibited decreased levels. KEGG pathway analysis of these 87 differentially accumulated metabolites revealed enrichment in phenylpropanoid, flavonoid, flavonol, and sesquiterpene biosynthesis pathways. In our study, we identified that the biosynthesis of phenylpropanoids, flavonoids and flavonols, and sesquiterpenes represented key metabolic pathways contributing to the variation in metabolites observed between PR and YW. Among these, the phenylpropanoid biosynthesis pathway was found to be particularly significant (Zhang et al., 2021), This pathway encompasses the production of a variety of compounds, including lignans, phenylpropanoids, flavonoids, coumarins, and other substances, and it serves as a source of precursors for many of the plant’s secondary metabolites. The phenylpropanoid pathway not only plays a pivotal role in plant growth and reproduction but also is involved in the plant’s defense mechanisms against both biotic and abiotic environmental stresses (Lavhale et al., 2018). Moreover, they contribute to plant reproduction by the accumulation of flavonol and anthocyanin pigments in floral parts (Rahim et al., 2023). Significance analysis of KEGG allowed the identification of the main biological functions of the different metabolites. This information provides a foundation for further biological studies on Farfarae Flos. The results of conjoint analysis with anti-inflammatory activity showed that 48 DAMs out of 87 DAMs were significantly correlated with the anti-inflammatory activity of Farfarae Flos, as evidenced by the inhibition of pro-inflammatory cytokines IL-6 and TNF-*α* and promotion of anti-inflammatory cytokine IL-10. It is worth mentioning that our study unveiled the potential anti-inflammatory activity of coumarins (e.g., Scopoletin) and sterols (e.g., Progesterone) in addition to the established role of flavonoids, alkaloids, and sesquiterpenes in Farfarae Flos.

Our study highlights the excellent qualities of PR, particularly its superior anti-inflammatory activity. Experimental evidence has consistently demonstrated higher content of indicator components and greater anti-inflammatory activity in PR compared to YW, and explores the underlying mechanisms contributing to the important quality characteristic ‘purplish-red better’ of Farfarae Flos. Our experimental results will guide the subsequent clinical use of Farfarae Flos, for instance, in the treatment of inflammation, PR should be considered the primary choice. We speculate that the synergistic accumulation of key active ingredients and the regulation of effective secondary metabolites during bud development may be the main reasons for the quality of Farfarae Flos. While this study focused on exploring the difference in anti-inflammatory activity between YW and PR through a combination of *in vivo*/*vitro* experiments and non-targeted metabolomics, and confirmed that these differences may be related to the accumulation of secondary metabolites in YW and PR, but failed to provide explanations for the reasons for the difference in anti-inflammatory activity in terms of signaling pathways and other underlying molecular mechanisms. These potential mechanisms will be clarified in our next study.

## 5 Conclusion

Our study compared the difference in anti-inflammatory activity and accumulation of secondary metabolites between two distinct colors of Farfarae Flos using a comprehensive approach that incorporated *in vivo*/*in vitro* experiments alongside metabolomics analysis. It was confirmed that both YW and PR could inhibit the release of NO, IL-6, and TNF-α, while enhancing the expression of IL-10. Furthermore, they exhibited the capacity to mitigate inflammation-induced liver and spleen cell damage, thereby exerting significant anti-inflammatory properties. Notably, PR exhibited superior anti-inflammatory activity compared to YW. These differences in anti-inflammatory activity were closely associated with the accumulation of plant secondary metabolites. Our findings may provide the experimental basis for the clinical application of Farfarae Flos, and have promising implications for the selection of varieties and quality evaluation of Farfarae Flos.

## Data Availability

The raw data supporting the conclusion of this article will be made available by the authors, without undue reservation.
