# Supplementary material for: Comparison of the difference in the anti-inflammatory activity of two different color types of Farfarae Flos based on in vitro, in vivo experiments and untargeted metabolomics
Source: Front Pharmacol. 2024 Sep 24;15:1463864. doi: 10.3389/fphar.2024.1463864 (PMC11459686; doi:10.3389/fphar.2024.1463864)
Supplement: Supplementary file 1 [file DataSheet1.pdf]

Supporting Information

Comparison of the difference in the anti-inflammatory activity of two different color types of *FARFARAE FLOS* based on *in vitro*, *in vivo* experiments and untargeted metabolomics

Kexin Zhou[a, b], Liang Peng[a, b], Yiyao Jing[a, b], Yao Luo[a, b], Yonggang Yan[a, b], Gang Zhang[a, b], Qi Guo [a, b], Bingyue Yang[a, b, *]

[a] *Shaanxi Qinling Application Development and Engineering Center of Chinese Herbal Medicine, College of Pharmacy, Shaanxi University of Chinese Medicine, Xi'an, 712046, China*

[b] *Key Laboratory for Research of 'Qin medicine' of Shaanxi Administration of Traditional Chinese Medicine, Xi'an, 712046, China*

[*] To whom correspondence should be addressed. Tel: +86-18064331298. Fax: +86-29-38185168. E-mail: 304951774@qq.com

Contents

- **Methods**
- **Figure S1.** The Base Peak Chromatogram (BPC) of positive ions
- **Figure S2.** The Base Peak Chromatogram (BPC) of negative ions
- **Figure S3.** Quality control (QC) and quality assurance(QA) of positive ions
- **Figure S4.** Quality control (QC) and quality assurance(QA) of negative ions
- **Figure S5.** The box plot and histogram of Quercetin 3-O-beta-D-glucosyl-(1->2)-beta-D-glucoside
- **Figure S6.** The box plot and histogram of Phloretin
- **Figure S7.** The box plot and histogram of Kaempferol
- **Figure S8.** The box plot and histogram of Isoquercitrin
- **Figure S9.** The box plot and histogram of Astragalin
- **Figure S10.** The box plot and histogram of Afzelin
- **Figure S11.** The box plot and histogram of Scopoletin
- **Figure S12.** The box plot and histogram of Progesterone
- **Figure S13.** The box plot and histogram of Polhovolide
- **Figure S14.** The box plot and histogram of Hypoxanthine
- **Figure S15.** The box plot and histogram of (E)-3-(4-Hydroxyphenyl)-2-propenal
- **Figure S16.** The box plot and histogram of Androsterone glucuronide
- **Figure S17.** The heatmap of 281 differential metabolites in YW and PR
- **Figure S18.** Correlation heat map of 281 differential metabolites in YW and PR
- **Figure S19.** Identification chart of 48 DAMs in YW and PR
- **Table 1.** Information on the relationship between 48 DAMs and inflammatory factors

Methods

Solvents and chemicals: Lipopolysaccharide (LPS) was purchased from Solarbio Science & Technology co. ltd. (Beijing, China). Mouse IL-6 ELISA Kit, TNF-α and IL-10 ELISA kit were purchased from Boster Biological Technology co. ltd. NO content detection kit was obtained from Grace Biotechnology co. ltd (Suzhou, China). Diclofenac Sodium Sustained Release Tablets were purchased from Sinopharm Zhijun Pharmaceutical co., ltd (Shenzhen, China). All the reagents were analytical grade and used directly without further purification.

Biocompatibility test of YW and PR: After being co-incubated for another 24 h, the medium was removed and 10 μL of alamarBlue® reagent in 100 μL complete growth medium was then added into each well. The plate was incubated for 4 h in a humidified incubator containing 5% CO_2 at 37°C. After that, 100 μL of the medium in each well was transferred into a 96-well black plate (Costar). Fluorescence was recorded using 560 nm as the excitation wavelength and 600 nm as the emission wavelength by using a microplate reader (Molecular Devices) according to the manufacturer's instructions. Cells seeded on the plate without YW and PR served as the TCP group. Tests were repeated four times for each group.

$$\text{Cell viability (\%)} = \frac{A_{sample} - A_{Blank}}{A_{TCP} - A_{Blank}} \times 100\%$$

While A_{sample} represented the absorbance of different samples; A_{TCP} represented the absorbance of the TCP group. A_{Blank} represented the absorbance of a complete growth medium containing 10% alamarBlue® reagent.

Metabolite extraction for the untargeted metabolomic analysis: Five samples of about 1 g each were taken from each of the two groups of *FARFARAE FLOS*, labeled yellowish-white 1-5 for the YW and purplish-red group 1-5 for the PR, wrapped in tin

foil and stored in liquid nitrogen for quick-freezing until use. Take the appropriate amount of samples (50 mg) in a 2 mL EP tube, and add 0.6 mL 2-chlorophenylalanine (4 ppm) methanol (-20 ℃), vortex for 30 s. Add 100 mg glass beads, put them into the tissue grinder, and grind for 60 s at 55 Hz. Room temperature ultrasound for 15 min. Centrifugation at 12000 rpm at 4 ℃ for 10 min, take 300 μL supernatant and filter through 0.22 μm membrane and add the filtrate into the detection bottle. Take 20 μL from each sample to the quality control (QC) samples; (These QC samples were used to monitor deviations of the analytical results from these pool mixtures and compare them to the errors caused by the analytical instrument itself). Use the rest of the samples for LC-MS detection.

UPLC-MS/MS analysis for the untargeted metabolomics: Chromatographic separation was used with an ACQUITY UPLC HSS T3 (150 × 2.1 mm, 1.8 μm, Waters) column maintained at 40 ℃. The temperature of the autosampler was 8 ℃. Gradient elution of analytes was carried out with 0.1% formic acid in water (C) and 0.1% formic acid in acetonitrile (D) or 5 mM ammonium formate in water (A) and acetonitrile (B) at a flow rate of 0.25 mL/min. Injection of 2 μL of each sample was done after equilibration. An increasing linear gradient of solvent B (v/v) was used as follows: 0~1 min, 2% B/D; 1~9 min, 2%~50% B/D; 9~12 min, 50%~98% B/D; 12~13.5 min, 98% B/D; 13.5~14 min, 98%~2% B/D; 14~20 min, 2% Dpositive model (14~17 min, 2% B-negative model).The ESI-MSn experiments were used with the spray voltage of 3.5 kV and -2.5 kV in positive and negative modes, respectively. Sheath gas and auxiliary gas were set at 30 and 10 arbitrary units, respectively. The capillary temperature was 325 ℃. respectively. The Orbitrap analyzer scanned over a mass range of m/z 100-1000 for the full scan at a mass resolution of 60 000. Data-dependent acquisition (DDA) MS/MS experiments were performed with HCD

scan. The cracking rate is 30%. Dynamic exclusion was implemented to remove some unnecessary information in MS/MS spectra.

Bioinformatic analysis of the untargeted metabolomic dataset: The raw data obtained were converted to mzXML format (xcms input file format) by Proteowizard software (v3.0.8789) and the acquired MS data were pre-processed using XCMS software, including peak picking, peak grouping, retention time correction, second peak grouping and annotation of isotopes and adducts. The LC-MS data in mz.XML format was processed by the XCMS, CAMERA and metaX toolboxes implemented using R software. Each ion is identified by retention time (RT) and m/z data. The intensity of each peak was recorded and a three-dimensional matrix containing any specified peak index (retention time - m/z pairs), sample name (observations) and ion intensity information (variables) was generated, and the data was batch normalized for peak area to allow comparison between different magnitudes of data. The online KEGG, HMDB database is used to annotate metabolites by matching the exact molecular weight data (m/z) of the sample to the data in the database. If the mass difference between observed and database values is less than 10 ppm, the metabolite is annotated and the molecular formula of the metabolite is further identified and validated by isotopic distribution measurements. In addition, an in-house library of metabolite fragment profiles was used to validate metabolite identification.

Results

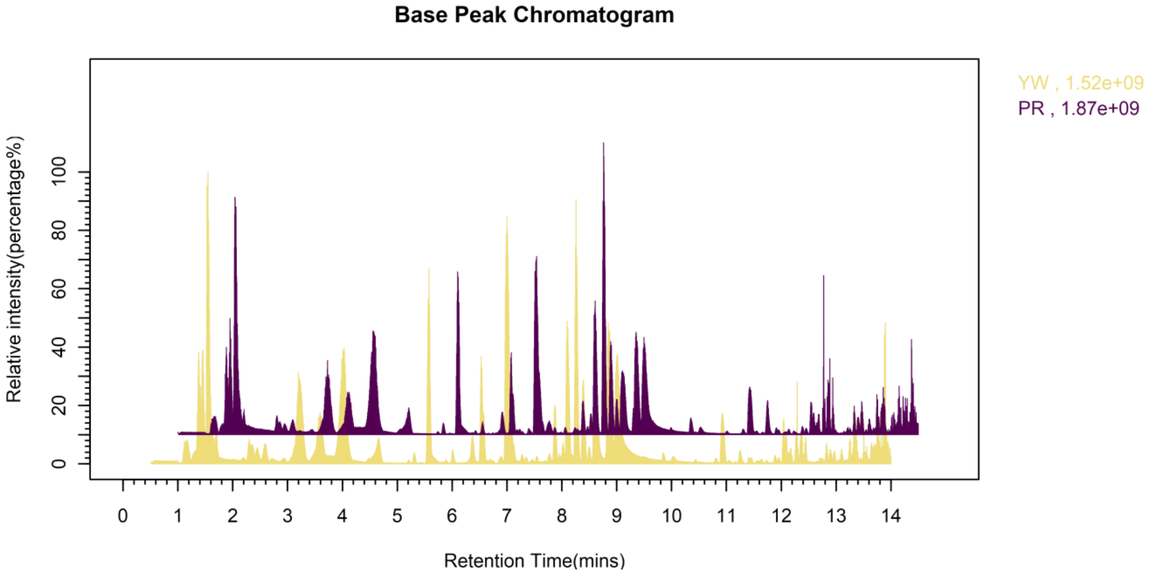

Figure S1. The Base Peak Chromatogram of positive ions

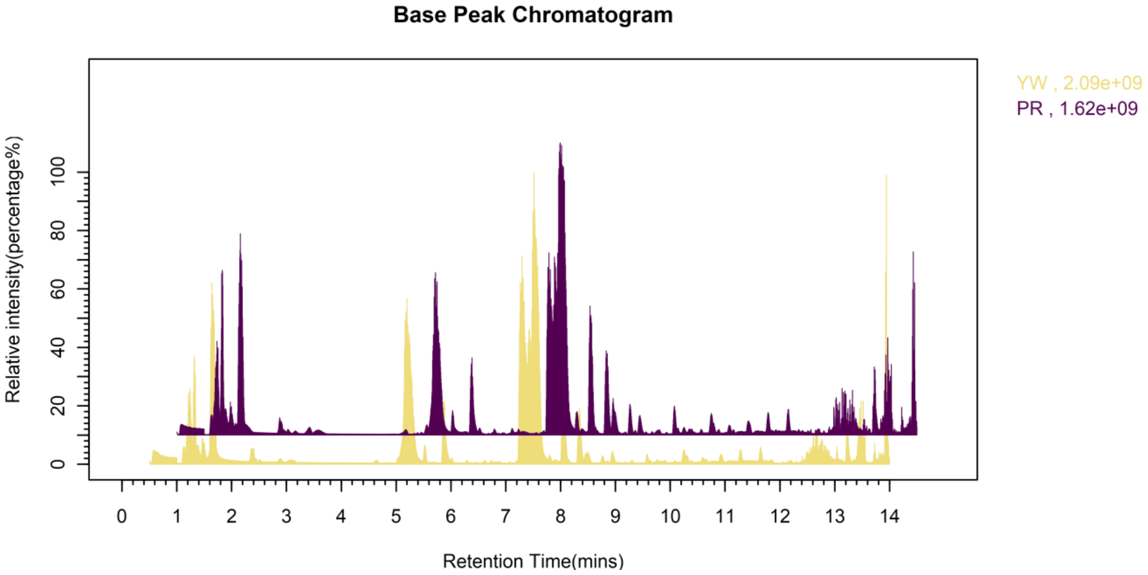

Figure S2. The Base Peak Chromatogram of negative ions

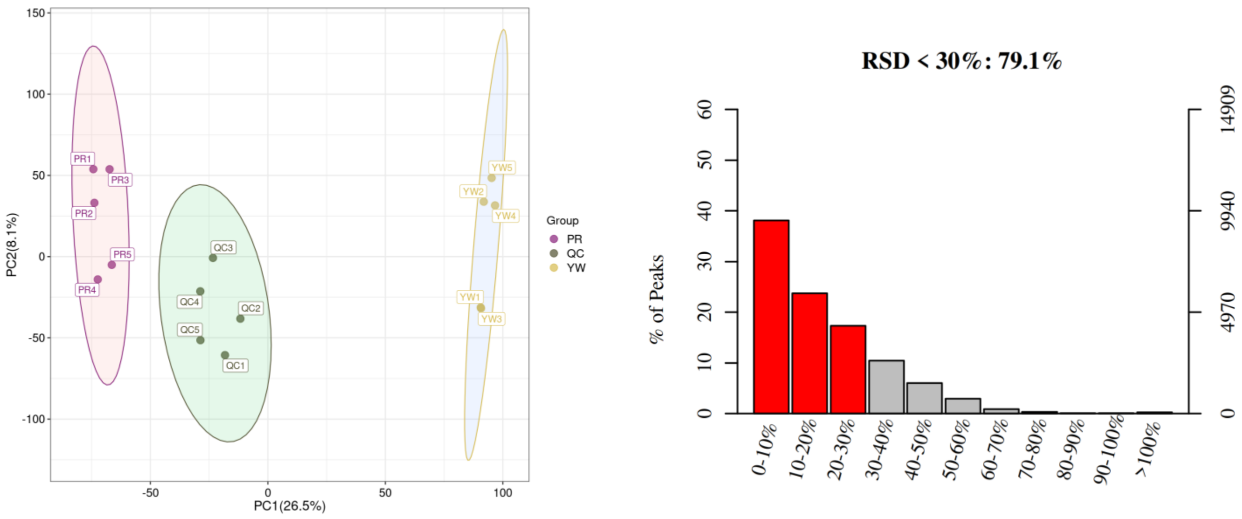

Figure S3. Quality control(QC) and quality assurance(QA) of positive ions

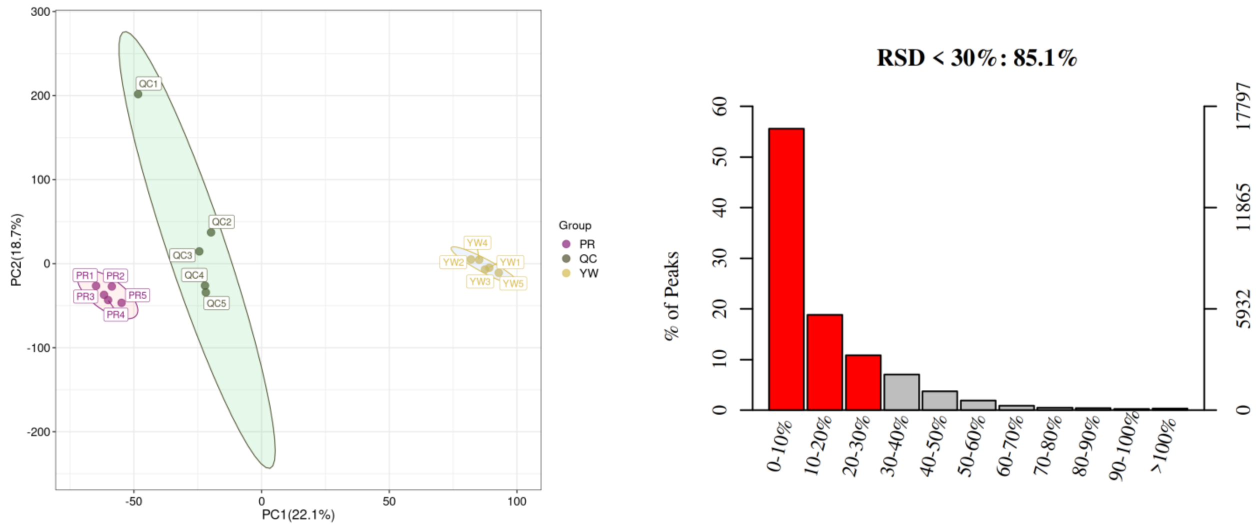

Figure S4. Quality control(QC) and quality assurance(QA) of negative ions

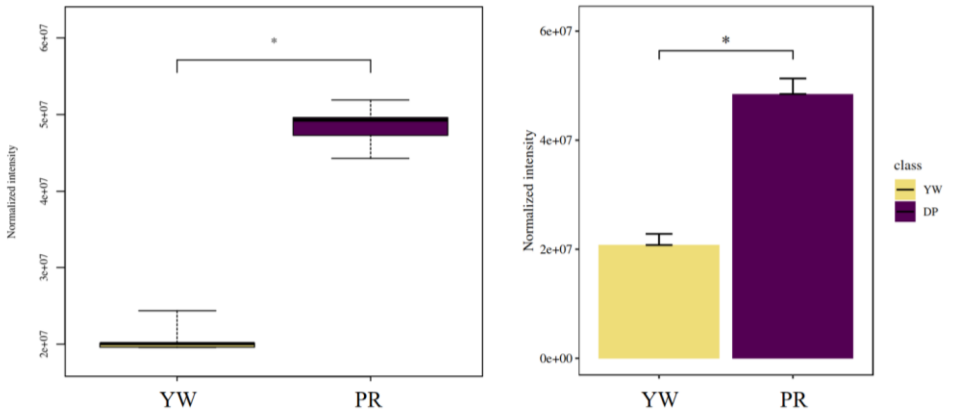

Figure S5. The box plot and histogram of Quercetin 3-O-beta-D-glucosyl-(1->2)-beta-D-glucoside

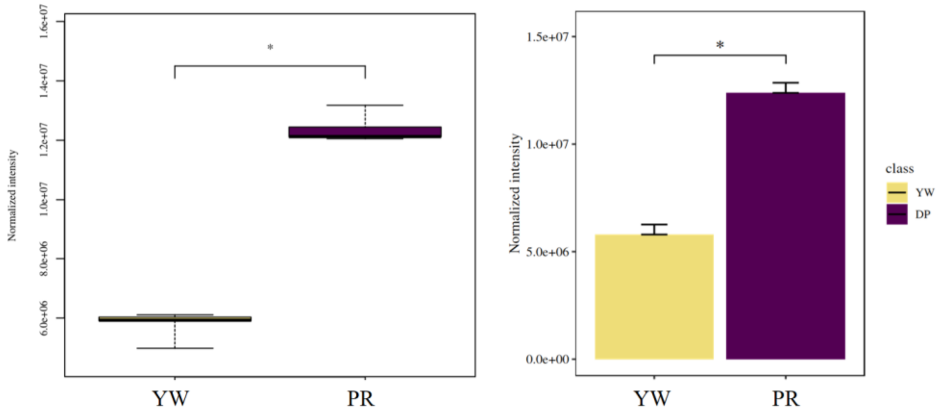

Figure S6. The box plot and histogram of Phloretin

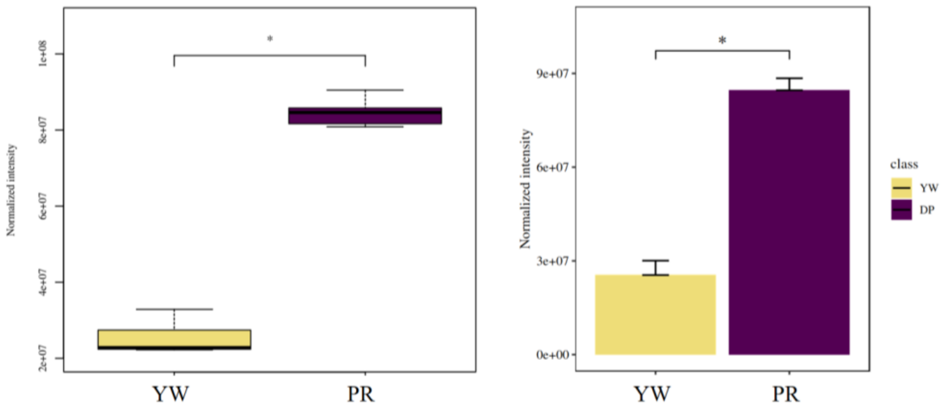

Figure S7. The box plot and histogram of Kaempferol

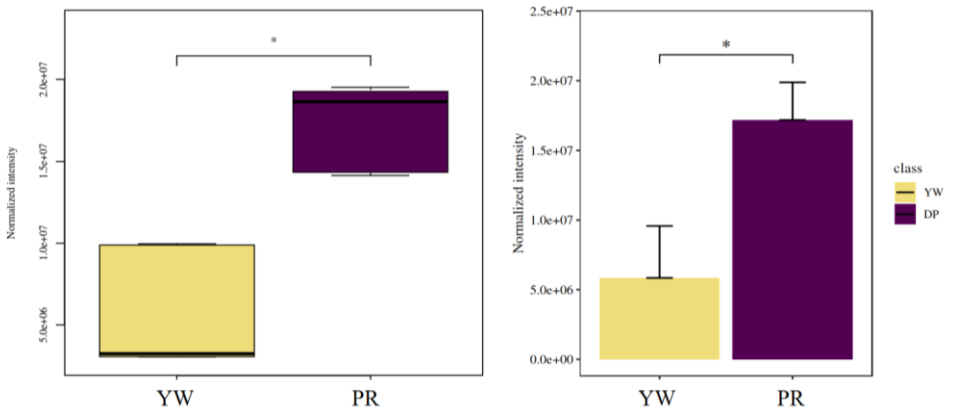

Figure S8. The box plot and histogram of Isoquercitrin

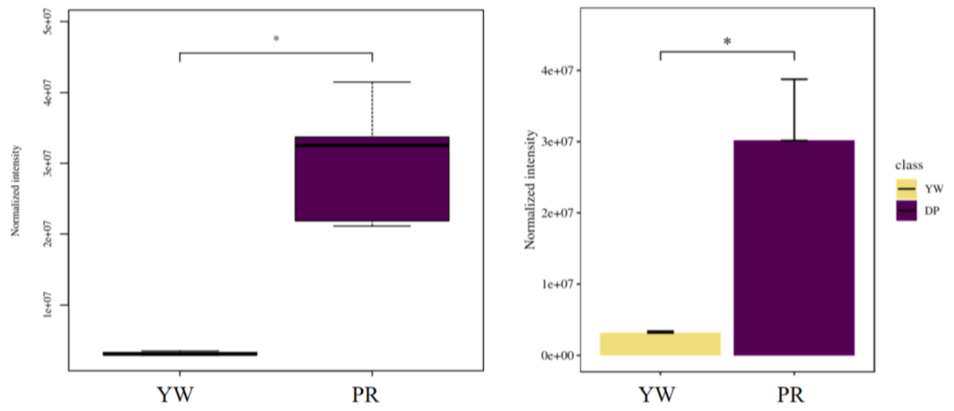

Figure S9. The box plot and histogram of Astragalin

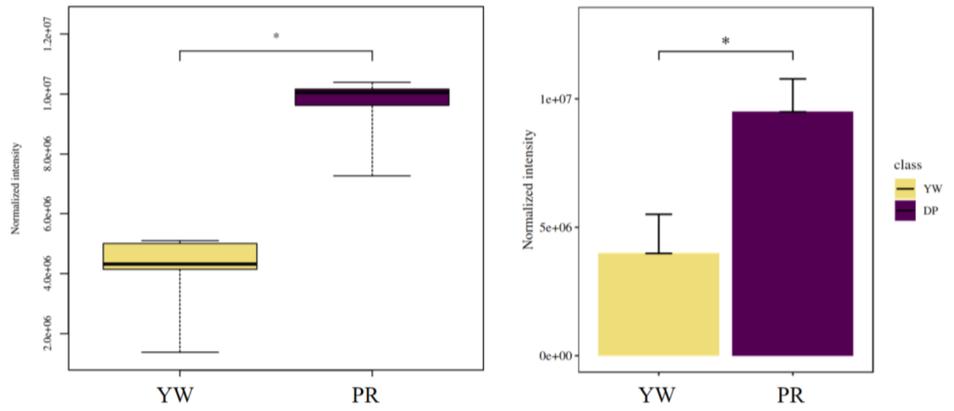

Figure S10. The box plot and histogram of Afzelin

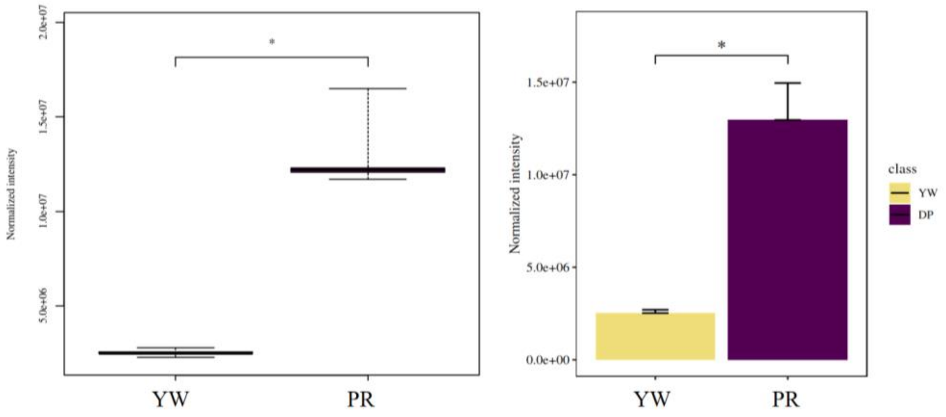

Figure S11. The box plot and histogram of Scopoletin

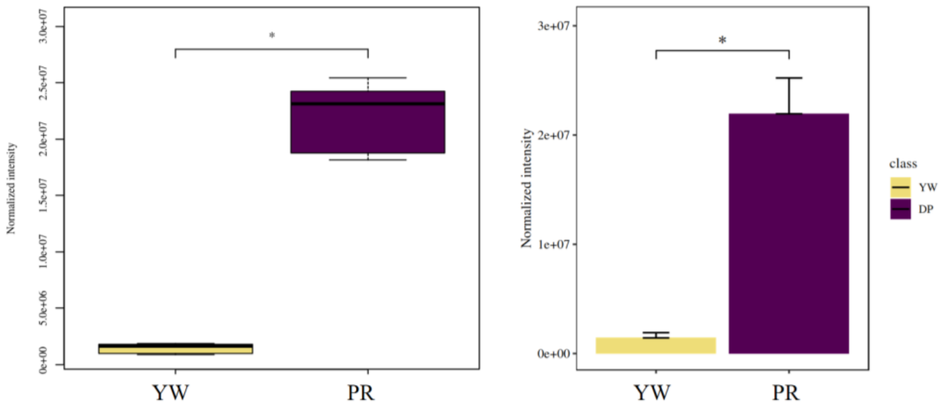

Figure S12. The box plot and histogram of Progesterone

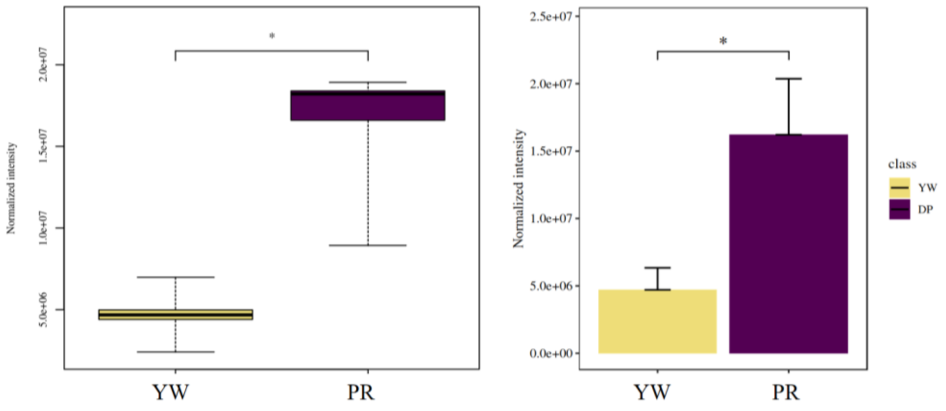

Figure S13. The box plot and histogram of Polhovolide

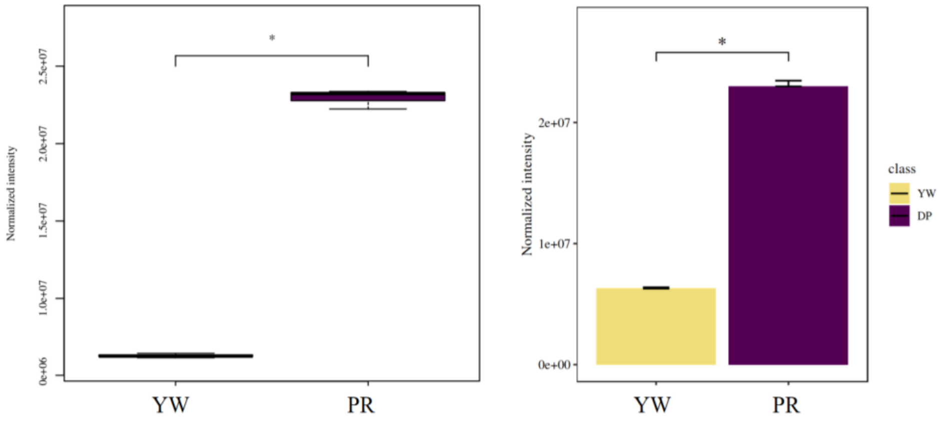

Figure S14. The box plot and histogram of Hypoxanthine

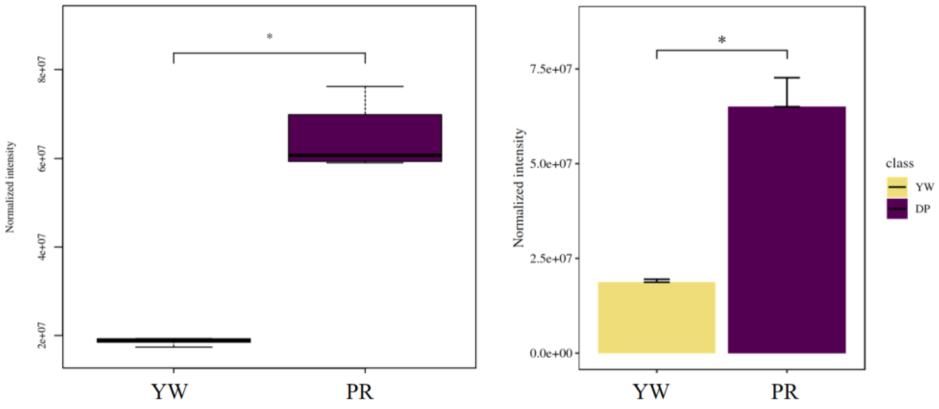

Figure S15. The box plot and histogram of (E)-3-(4-Hydroxyphenyl)-2-propenal

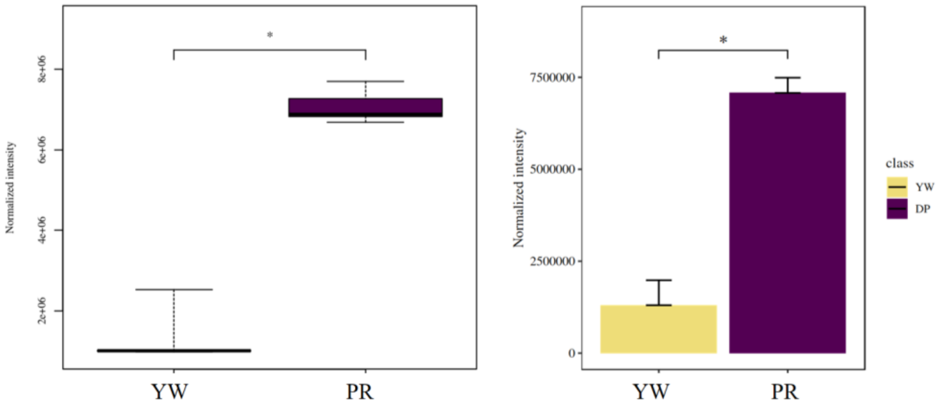

Figure S16. The box plot and histogram of Androsterone glucuronide

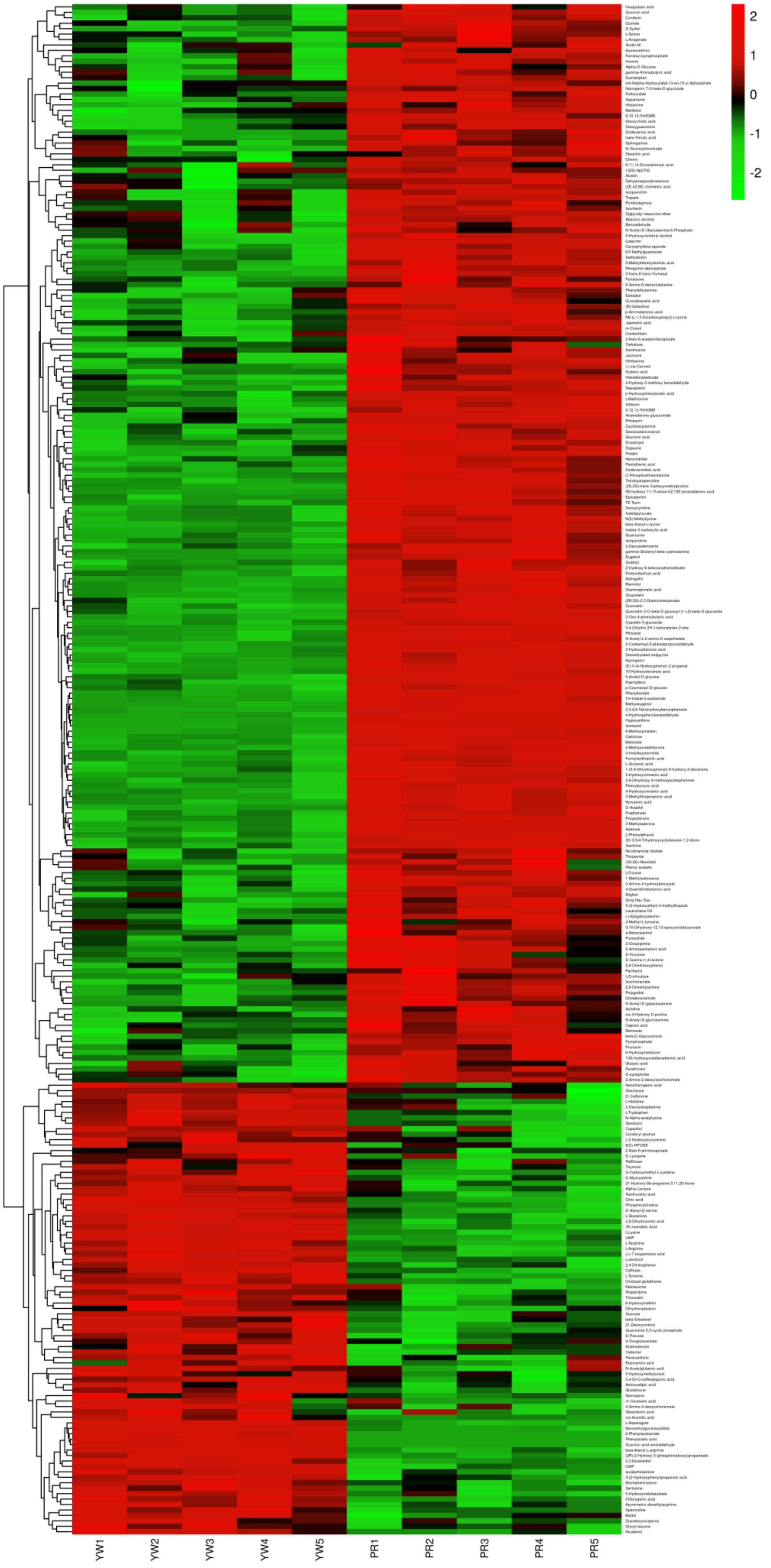

Figure S17. The heat map of 281 differential metabolites in YW and PR

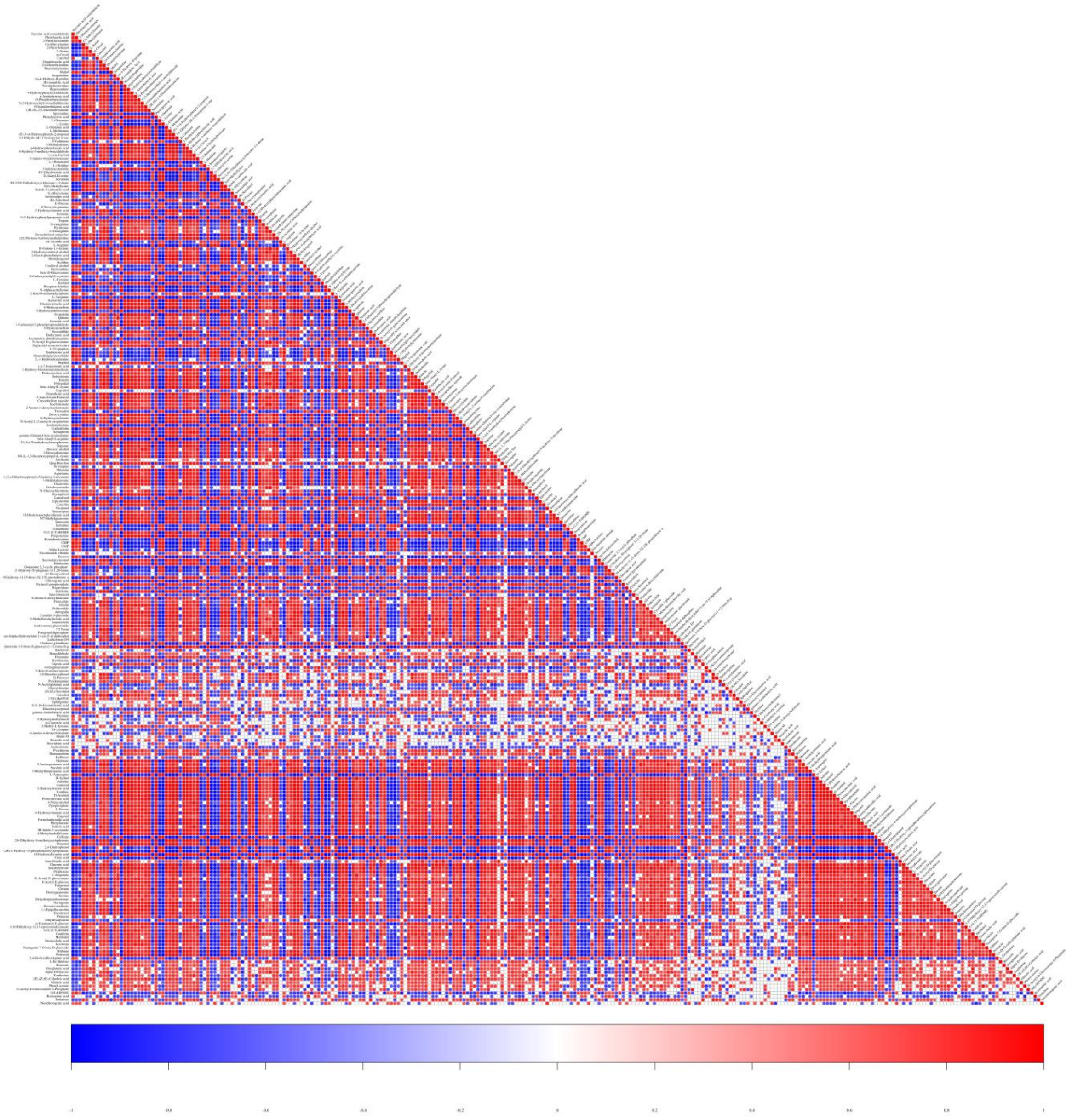

Figure S18. Correlation heat map of 281 differential metabolites in YW and PR

p-Hydroxyphenylacetic acid

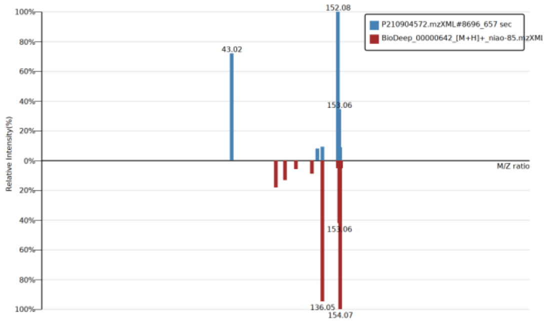

(E)-3-(4-Hydroxyphenyl)-2-propenal

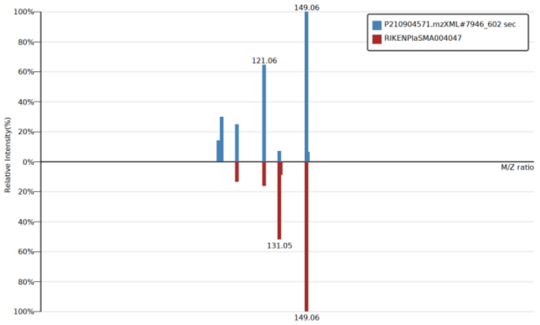

Catechin

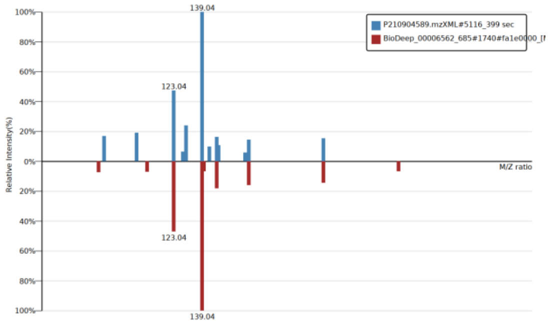

Isoniazid

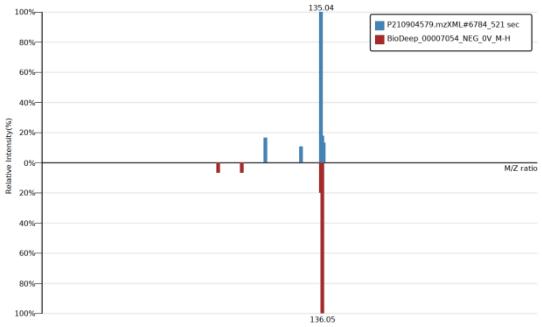

Tropate

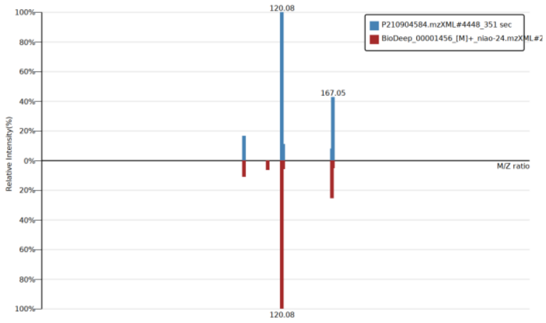

2-trans,6-trans-Farnesal

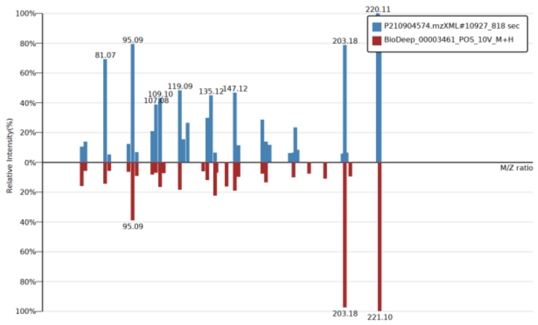

Diglycidyl resorcinol ether

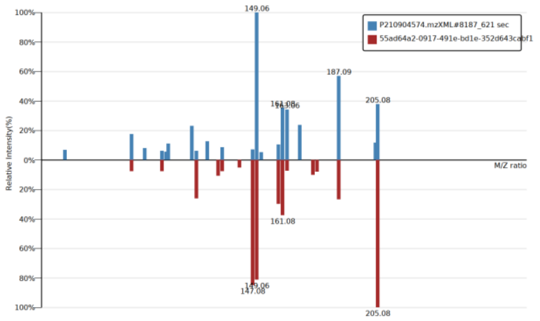

Caryophyllene epoxide

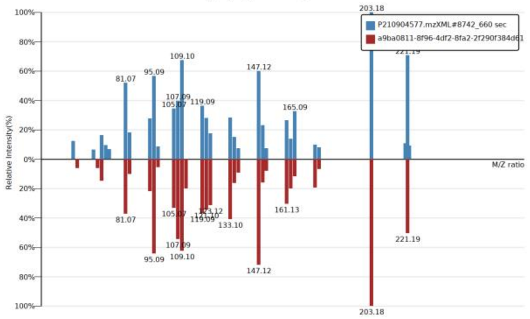

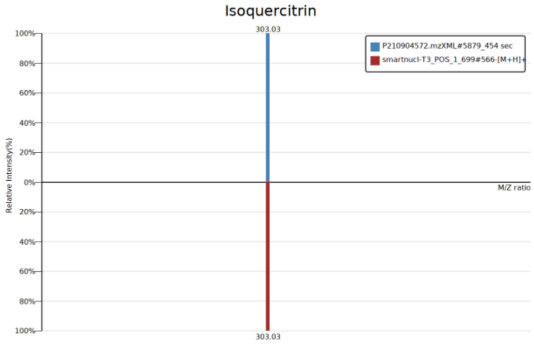
Isoquercitrin
P210904572.mzXML#5879_454 sec
smartnuci-T3_POS_1_699#566-[M+H]+
Relative Intensity(%)
M/Z ratio

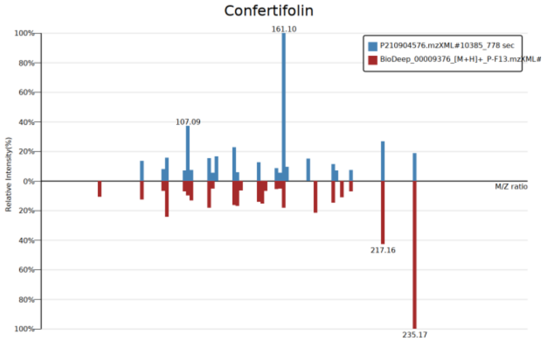
Confertifolin
P210904576.mzXML#10385_778 sec
BioDeep_00009376_[M+H]+_P-F13.mzXML#
Relative Intensity(%)
M/Z ratio

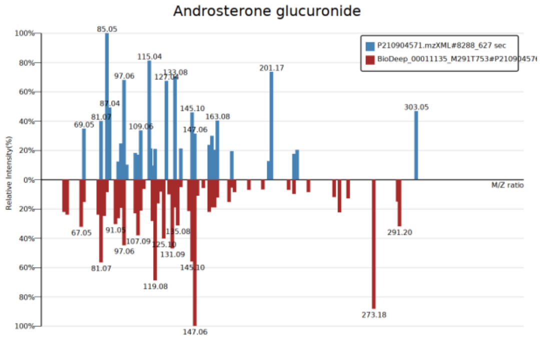
Androsterone glucuronide
P210904571.mzXML#8288_627 sec
Relative Intensity(%)
M/Z ratio

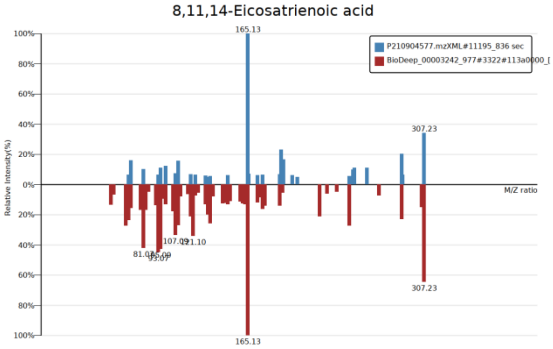
8,11,14-Eicosatrienoic acid
P210904577.mzXML#11195_836 sec
Relative Intensity(%)
M/Z ratio

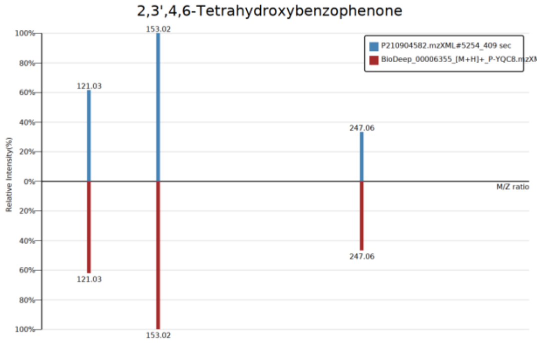
2,3',4,6-Tetrahydroxybenzophenone
P210904582.mzXML#5254_409 sec
Relative Intensity(%)
M/Z ratio

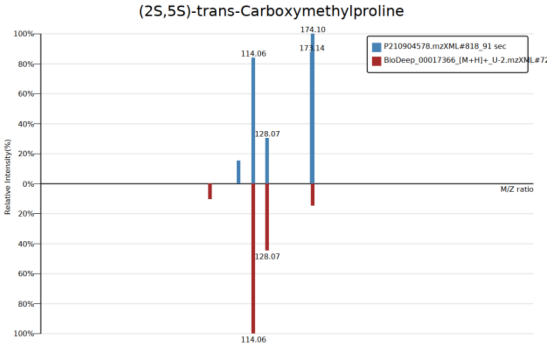
(2S,5S)-trans-Carboxymethylproline
P210904578.mzXML#818_91 sec
Relative Intensity(%)
M/Z ratio

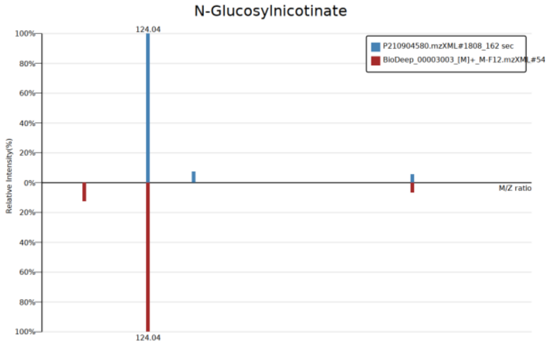
N-Glucosylnicotinate
P210904580.mzXML#1808_162 sec
Relative Intensity(%)
M/Z ratio

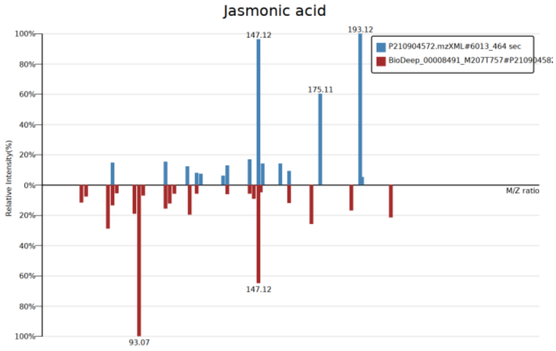
Jasmonic acid
P210904572.mzXML#6013_464 sec
Relative Intensity(%)
M/Z ratio

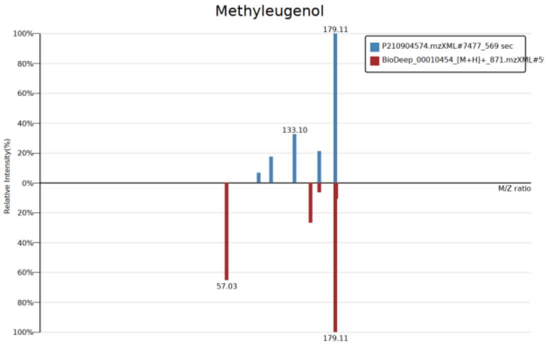
Methyleugenol
P210904574.mzXML#7477_569 sec
179.11
133.10
57.03
179.11
Relative Intensity(%)
M/Z ratio

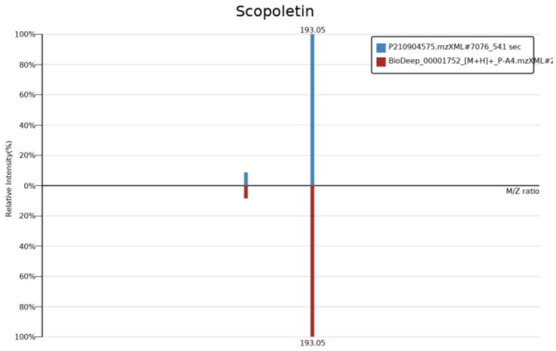
Scopoletin
P210904575.mzXML#7076_541 sec
193.05
193.05
Relative Intensity(%)
M/Z ratio

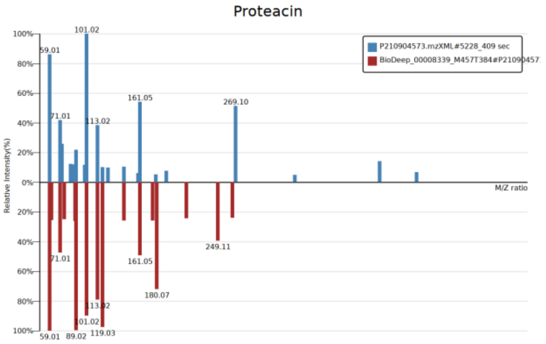
Proteacin
P210904573.mzXML#5228_409 sec
101.02
59.01
161.05
269.10
71.01
71.01
161.05
249.11
180.07
59.01
89.02
119.03
Relative Intensity(%)
M/Z ratio

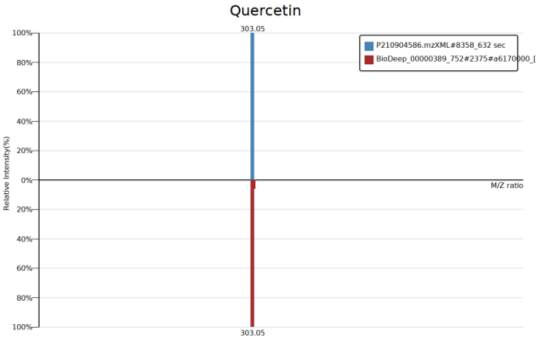
Quercetin
P210904586.mzXML#8358_632 sec
303.05
303.05
Relative Intensity(%)
M/Z ratio

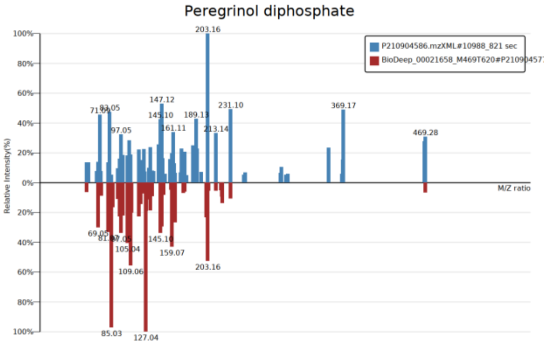
Peregrinol diphosphate
P210904586.mzXML#10988_821 sec
203.16
147.12
231.10
369.17
469.28
97.05
161.11
213.14
145.10
159.07
109.06
203.16
85.03
127.04
Relative Intensity(%)
M/Z ratio

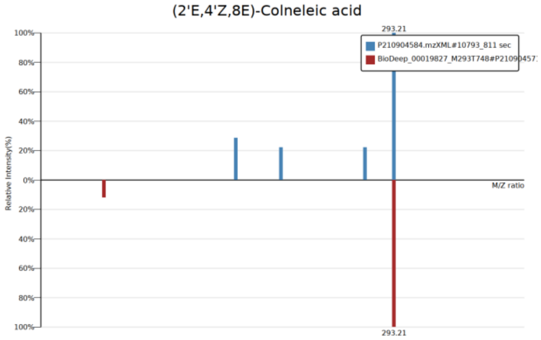
(2'E,4'Z,8E)-Colneleic acid
P210904584.mzXML#10793_811 sec
293.21
293.21
Relative Intensity(%)
M/Z ratio

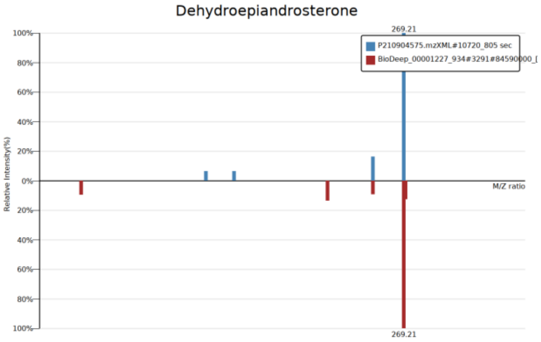
Dehydroepiandrosterone
P210904575.mzXML#10720_805 sec
269.21
269.21
Relative Intensity(%)
M/Z ratio

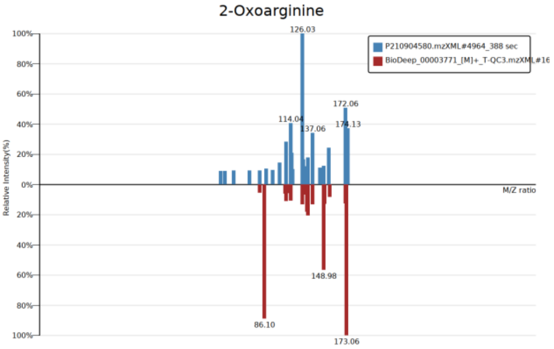
2-Oxoarginine
P210904580.mzXML#4964_388 sec
126.03
172.06
137.06
148.98
86.10
173.06
Relative Intensity(%)
M/Z ratio

Phloretin

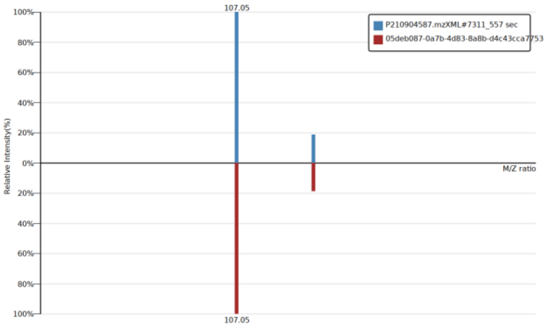

5-Methyltetrahydrofolic acid

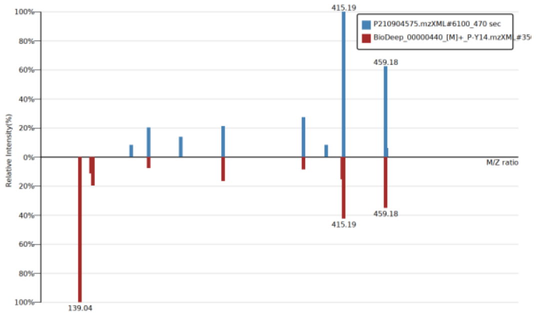

Progesterone

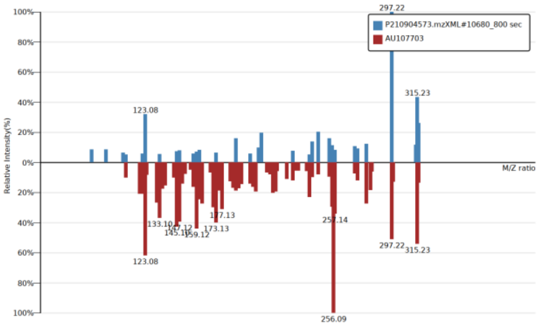

2-Oxo-4-phenylbutyric acid

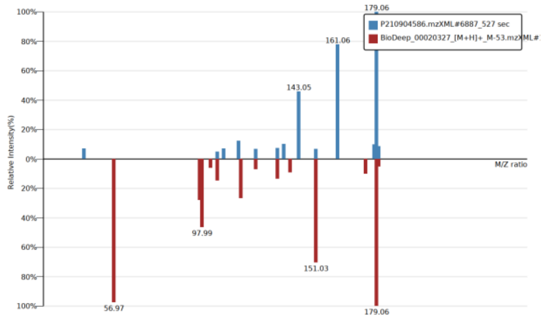

Cetirizine

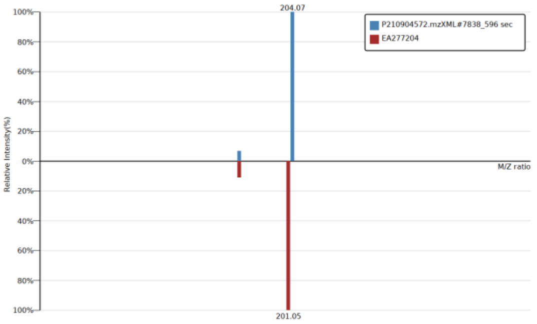

5-Aminopentanoic acid

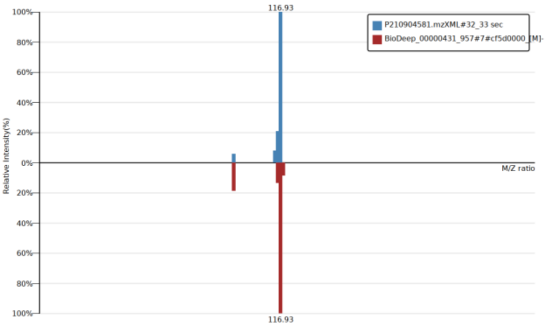

cis-4-Hydroxy-D-proline

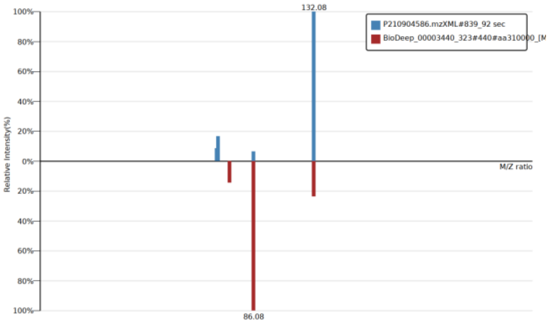

Citrinin

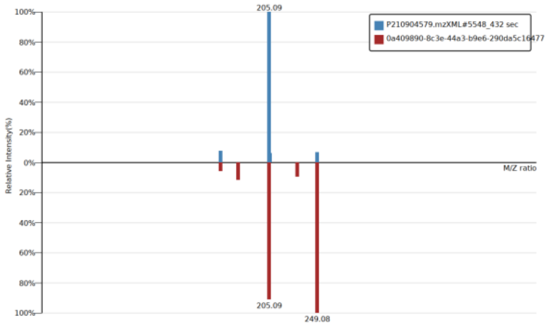

Hypoxanthine

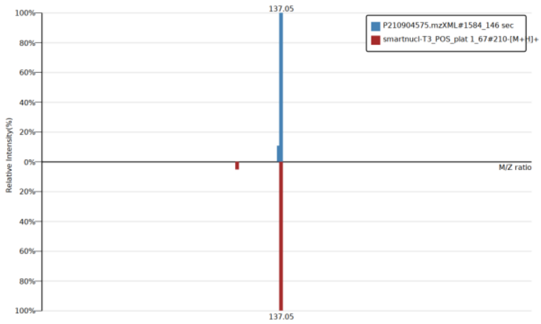

Cyanidin 3-glucoside

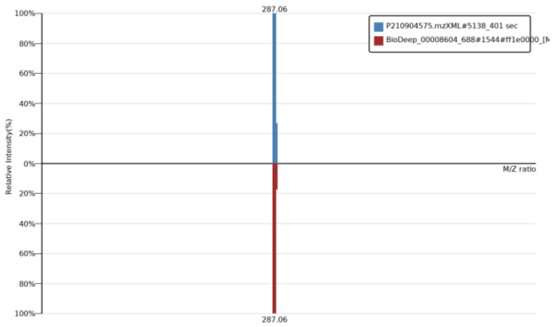

Kaempferol

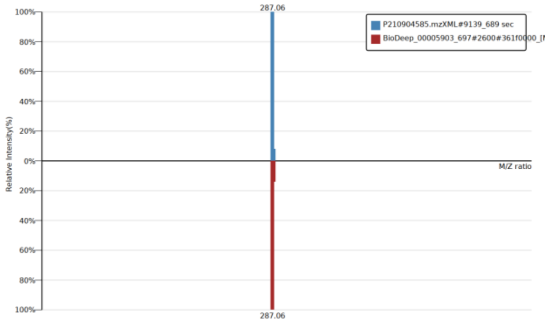

Illudin M

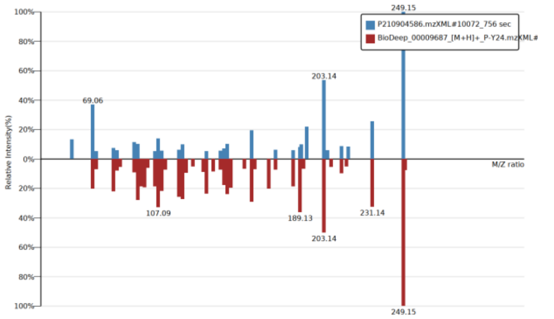

Polhovolide

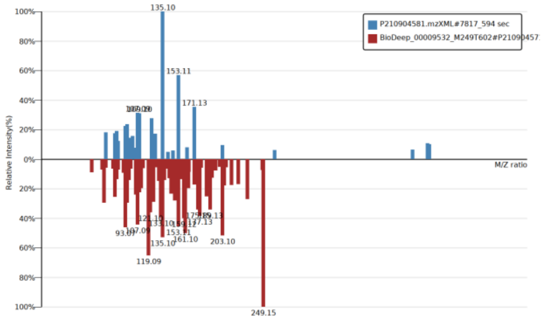

4-Hydroxyphenylacetaldehyde

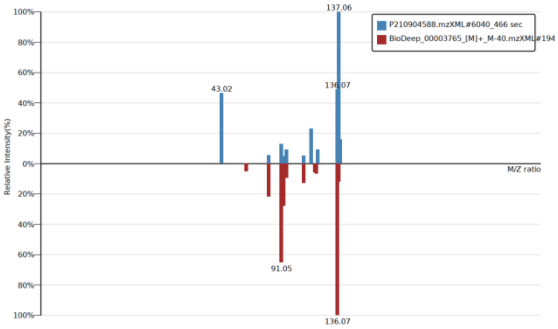

Astragalin

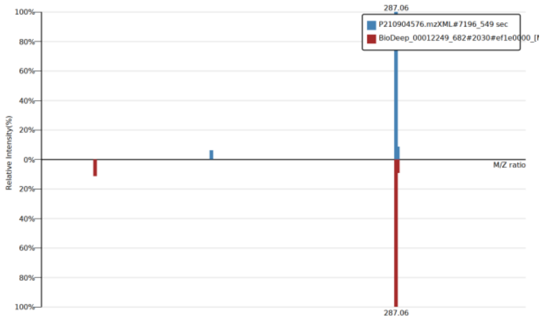

Eugenol

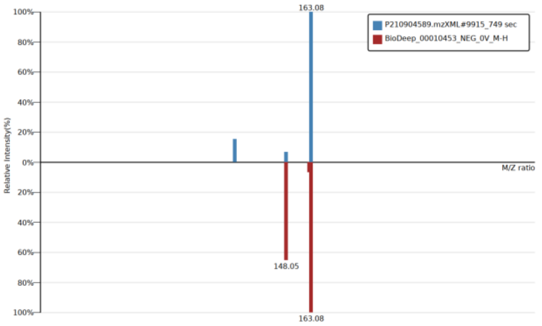

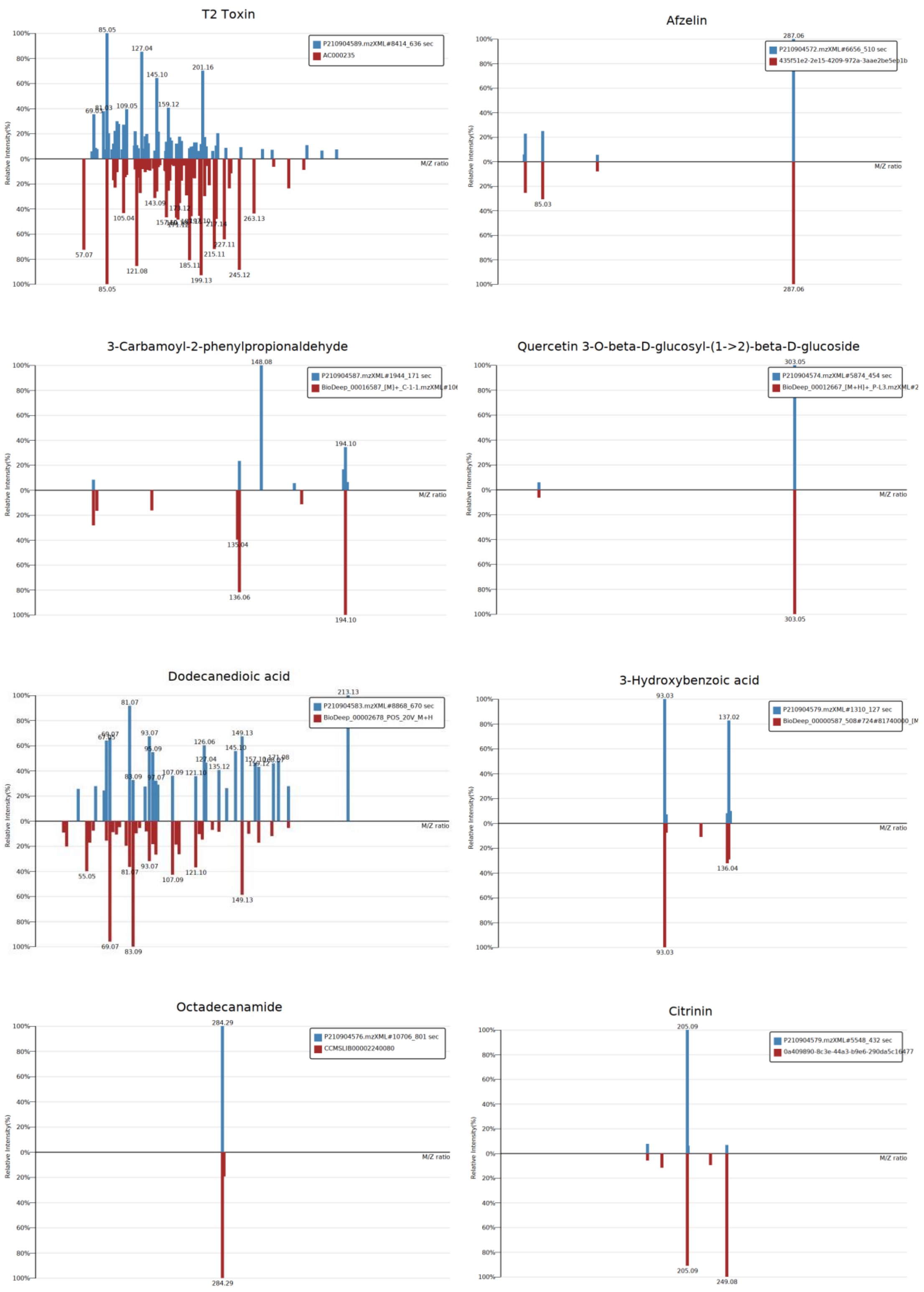

Figure S19. Identification chart of 48 DAMs in YW and PR

Table 1. Information on the relationship between 48 DAMs and inflammatory factors

Compounds	Related Inflammatory Factors	Relation
(2E,4Z,8E)-Colneleic acid	IL-6	negative
(2S,5S)-trans-Carboxymethylproline	IL-6	negative
	THF-α	negative
(E)-3-(4-Hydroxyphenyl)-2-propenal	IL-10	positive
	IL-6	negative
	THF-α	negative
2,3,4,6-Tetrahydroxybenzophenone	THF-α	negative
	IL-6	negative
2-Oxo-4-phenylbutyric acid	IL-10	positive
	IL-6	negative
2-Oxoarginine	IL-6	negative
2-trans,6-trans-Farnesal	THF-α	negative
3-Carbamoyl-2-phenylpropionaldehyde	IL-10	positive
	THF-α	negative
3-Hydroxybenzoic acid	IL-10	positive
	THF-α	negative
4-Hydroxyphenylacetaldehyde	IL-10	positive
	IL-6	negative
5-Aminopentanoic acid	IL-10	positive
	IL-6	negative
5-Hydroxyconiferyl alcohol	IL-6	negative
	THF-α	negative
5-Methyltetrahydrofolic acid	IL-6	negative
8,11,14-Eicosatrienoic acid	IL-10	positive
	THF-α	negative
Afzelin	IL-10	positive

	THF-α	negative
	IL-6	negative
	IL-10	positive
Androsterone glucuronide	THF-α	negative
	IL-6	negative
	IL-10	positive
Astragalin	THF-α	negative
	IL-6	negative
Caryophyllene epoxide	THF-α	negative
Catechin	THF-α	negative
	IL-6	negative
Cetirizine	THF-α	negative
	IL-6	negative
cis-4-Hydroxy-D-proline	IL-10	positive
	IL-6	negative
Citrinin	IL-6	negative
	THF-α	negative
Confertifolin	THF-α	negative
Cyanidin 3-glucoside	IL-10	positive
	IL-6	negative
Dehydroepiandrosterone	THF-α	negative
	IL-6	negative
Diglycidyl resorcinol ether	IL-6	negative
	THF-α	negative
Dodecanedioic acid	THF-α	negative
Eugenol	THF-α	negative
	IL-6	negative
Hypoxanthine	IL-10	positive

	THF-α	negative
	IL-6	negative
Illudin M	IL-10	positive
Isoniazid	IL-10	positive
	THF-α	negative
Isoquercitrin	IL-10	positive
	THF-α	negative
	IL-6	negative
Jasmonic acid	IL-6	negative
	THF-α	negative
Kaempferol	IL-10	positive
	THF-α	negative
	IL-6	negative
Methyleugenol	THF-α	negative
	IL-6	negative
N-Glucosylnicotinate	THF-α	negative
Octadecanamide	IL-10	positive
	THF-α	negative
Peregrinol diphosphate	THF-α	negative
	IL-6	negative
Phloretin	IL-10	positive
	IL-6	negative
	THF-α	negative
p-Hydroxyphenylacetic acid	THF-α	negative
Polhovolide	IL-10	positive
	THF-α	negative
	IL-6	negative
Progesterone	IL-10	positive

	IL-6	negative
	THF-α	negative
Proteacin	IL-6	negative
Quercetin	THF-α	negative
	IL-6	negative
Quercetin 3-O-beta-D-glucosyl-(1->2)-beta-D-glucoside	IL-10	positive
	THF-α	negative
	IL-6	negative
Scopoletin	IL-10	positive
	THF-α	negative
	IL-6	negative
T2 Toxin	IL-10	positive
	THF-α	negative
Tropate	THF-α	negative
